# Metformin and weight loss medication impact on survival outcomes in older women with obesity-related cancers

**DOI:** 10.1038/s41598-025-09393-1

**Published:** 2025-07-01

**Authors:** Maryam R. Hussain, Omer Abdelgadir, Yong-Fang Kuo, Steven Canfield, David S. Lopez

**Affiliations:** 1 School of Public and Population Health, University of Texas Medical Branch, Galveston, TX 77555 USA; 2 Department of Internal Medicine, University of New Mexico Health Sciences Center, Albuquerque, NM 87131 USA; 3 Graduate School of Biomedical Science, University of Texas Medical Branch, 301 University Blvd, Galveston, TX 77555 USA; 4 O’Donnell School of Public Health, University of Texas Southwestern Medical Center, Dallas, TX 75390 USA; 5 Department of Surgery, Division of Urology, UTHealth McGovern Medical School, Houston, TX 77030 USA; 6 Simmons Comprehensive Cancer Center, University of Texas Southwestern Medical Center, Dallas, TX 75390 USA

**Keywords:** Metformin, Weight loss medication, Breast cancer, Colorectal cancer, Ovarian cancer, Endometrial cancer, Medical research, Oncology, Cancer, Breast cancer, Cancer epidemiology, Gastrointestinal cancer, Gynaecological cancer

## Abstract

While the association between metformin and mortality in obesity-related cancers (ORC: breast (BrCa), colorectal (CRC), endometrial (ECa), and ovarian (OCa)) remains inconsistent, the impact of weight loss medication (WLM) on mortality in ORC is largely unexplored, particularly among older women. This study aims to investigate the association between pre-diagnostic use of metformin and WLM with all-cause and cancer-specific mortality in older women with ORC. A retrospective cohort of 63,907 women (≥ 65 years) with ORC was identified using SEER-Medicare 2007–2015 data. Pre-diagnostic prescriptions of metformin and WLM were ascertained, and inverse probability of treatment weighting using propensity score (IPTW-PS) was utilized to balance baseline patient characteristics. Cox proportional hazards and competing-risks models were conducted. Metformin was associated with all-cause- (HR:1.86; 95% CI: 1.81–1.92) and ORC-specific mortality (HR: 1.71; 95% CI: 1.63–1.77). Likewise, WLM was associated with all-cause- (HR:1.64; 95% CI: 1.59–1.71) and ORC-specific mortality (HR: 1.55; 95% CI: 1.48–1.62). Dual use of metformin and WLM was associated with all-cause- (HR:1.39; 95% CI: 1.34–1.45) and ORC-specific mortality (HR: 1.28; 95% CI: 1.21–1.53). Significant associations were observed in ORC subgroup analyses, except that WLM was inversely associated with ECa-specific morality. Pre-diagnostic metformin and WLM were associated with an increased mortality risk in older women with ORC, with effects more pronounced among metformin users. Further prospective studies are needed to substantiate these findings.

**Supplementary Information:**

The online version contains supplementary material available at 10.1038/s41598-025-09393-1.

## Introduction

Obesity has reached epidemic proportions in the United States, with over 36% of adults currently classified as obese. Severe obesity affects nearly 10%, with a higher prevalence in women than men^1^. Substantial health risks have been linked to obesity^2,3^. Older women with obesity are prone to a high risk of obesity-related cancer (ORC) including breast cancer (BrCa), colorectal cancer (CRC), endometrial cancer (ECa), and ovarian cancer (OCa)^4^. These cancers share complex etiologies, often involving chronic inflammation, insulin resistance, and hormonal dysregulation^2,4,5^. While the precise mechanisms linking obesity to ORC risk remain to be fully understood, the association is well-established^5,6^. The American Cancer Society’s 2024 report estimates 42,170 deaths from BrCa, 13,860 from ECa, and 12,730 from OCs in women^7^.

Weight management strategies, including lifestyle modifications and weight loss medication (WLM), are essential for addressing the obesity crisis^8^. Understanding the impact of weight management strategies on the survival outcomes of older women with ORC is of paramount importance. Effective weight management in this population necessitates careful consideration, as it is intertwined with the unique physiological and pharmacological challenges faced by aging individuals. Age-related changes, such as fat redistribution and sarcopenia, can significantly increase the risks associated with traditional weight loss approaches^9^. Moreover, the high prevalence of comorbidities in this population, coupled with polypharmacy, elevates the risk of drug-drug interactions, adverse events, and medication non-adherence^10^.

Metformin has been used off-label for weight loss purposes^11^ and has also been investigated for its potential anti-cancer properties^12^. Meta-analyses of observational studies and randomized controlled trials (RCTs) have reported associations between metformin use and reduced all-cause and cancer-specific mortality in patients with cancer^13–17^. However, these findings are not consistent. Some studies have reported increased mortality risks with metformin use^18,19^, while others have found no benefit. Notably, the MA.32 RCT found no improvement in invasive disease–free survival among non-diabetic breast cancer patients receiving metformin^20^. These conflicting results may be partly explained by methodological limitations in earlier observational studies, including immortal time bias, which can lead to inaccurate estimates of benefit or risk^21^.

WLMs, including diethylpropion, liraglutide, lorcaserin, orlistat, phendimetrazine, and phentermine, have gained prominence in obesity management due, in part, to their low cost^22^. While their efficacy in weight loss is evident, long-term effects on cancer outcomes remain unclear. Given the rising prevalence of obesity and the widespread use of these medications, rigorous safety evaluations are imperative. Notably, in early 2020, the US Food and Drug Administration (FDA) reported an increased risk of cancer linked to lorcaserin^23^. Although some studies suggested a connection between liraglutide and thyroid cancer, a meta-analysis failed to confirm this association^24–26^. Additionally, in vitro and in vivo studies have shown that orlistat may have anti-tumor properties in capacity in ECa, OCa, and CRC^27–29^.

Metformin may benefit cancer outcomes by directly inhibiting cancer cell proliferation^30^ and indirectly suppressing tumor progression through changes in the adipose tissue microenvironment and systemic endocrine alterations^31^. However, its potential negative impact include immunosuppressive effects, such as reduced proinflammatory cytokine production and promotion of regulatory T cell differentiation, which could impair immune responses to cancer^32,33^. Similarly, while the immunomodulatory effects of WLM are less understood, they are believed to impact the immune system indirectly through metabolic actions, particularly in lipid metabolism^34^.

Body weight has been shown to influence ORC survival outcomes. While excess weight is often detrimental^35–38^, the “obesity paradox” highlights potential exceptions^39^. Some studies have reported a paradoxical association in CRC and BrCa, where overweight or mildly obese cancer patients had better survival compared to normal-weight patients^40–43^. Given the substantial burden of ORC, the growing prevalence of obesity, the widespread use of metformin and WLM, and the limited knowledge about how these medications affect ORC survival in the geriatric population, this study aimed to investigate the association between pre-diagnostic use of metformin and WLM with all-cause and ORC-specific mortality in older women with ORC.

## Materials and methods

### Data

Data was obtained from the Surveillance, Epidemiology and End Results (SEER)-Medicare 2007–2015, a linkage of population-based cancer registries from 19 SEER regions with longitudinal Medicare administrative data^44^. Data on the cancer-free population was collected from the 5% non-cancer file. The SEER program collects detailed information on cancer cases, including demographics and clinical and survival information^45^. Medicare maintains comprehensive records of healthcare services for individuals aged 65 and older, including diagnoses, treatments, and health outcomes. The present study was approved by the Institutional Review Board of the University of Texas Medical Branch at Galveston, Texas, USA (IRB Approval Code: 20–0237; Approval date: 15 September 2020). Due to the retrospective nature of this study, no patient informed consent specific to this study was required.

### Study cohort

**Fig. 1:**
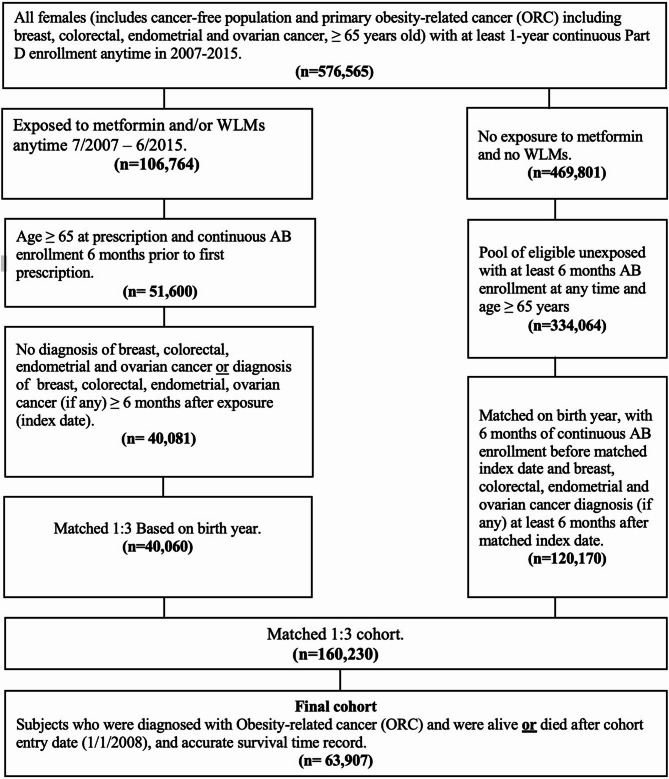
Cohort selection flowchart. Study inclusion and exclusion criteria.

This retrospective cohort study initially included all women aged ≥ 65 years (*n* = 576,565) with continuous Medicare Part D enrollment from 7/2007 to 6/2015. Participants were categorized as exposed or unexposed based on metformin or WLM prescriptions from 7/2007 to 6/2015. The study’s index date was defined as the date of initiation of metformin or WLM. Exposed subjects younger than 65 years at the index date, with less than six months of continuous Medicare Parts A and B enrollment prior to the index date, or with any ORC diagnosis including BrCa, CRC, ECa, or OCa (Supplemental Table 1) within six months of the index date were excluded. Potential unexposed matches were subjects aged ≥ 65 years with at least six months of Medicare Parts A and B coverage. Unexposed subjects were matched one to three with exposed subjects based on birth year and assigned the same index date (matched cohort *n* = 160,230). The final cohort (*n* = 63,907) comprised subjects diagnosed with ORC from 1/2008 to 12/2015. It was ensured that both exposed and unexposed groups maintained continuous Medicare Parts A and B enrollment for at least six months before the assigned index date, and the index date was at least six months before any diagnosis of ORC. Figure 1 and Supplemental Fig. 1 illustrate the cohort selection and study timeline. Fourteen subgroups based on tumor sidedness, stage, and grade were created for stratified analysis as indicated in Supplementary Fig. 2. For the subgroup analyses, advanced stage cancer was defined as the American Joint Committee on Cancer (AJCC) stages III and IV, while high-grade cancer was defined as Grade 3 (poorly differentiated) and Grade 4 (undifferentiated).

### Pre-diagnostic use of metformin and WLM

The pre-diagnostic use of metformin and WLM was the primary exposure. Prescription of metformin and WLM was established from the Medicare Part D file using National Drug Codes (NDC) and Current Procedural Terminology (CPT) codes (Supplemental Table 2). WLM included in this study were diethylpropion, liraglutide, lorcaserin, orlistat, phendimetrazine, and phentermine. The subjects were divided into four groups based on pre-diagnostic use of metformin and WLM: neither users (reference group), metformin alone, WLM alone, and dual users. The index date was defined as the date of initiation of metformin or WLM within the study period; for patients who used both metformin and WLM, at least six months between the latter of the two dates and ORC diagnosis (if any) was required. These criteria were also applied to the metformin and WLM groups. At least six months between the prescription date and the diagnosis of ORC (if any) was required to be classified as exposed. For dual users, a minimum of six months between the later of the two prescription dates and the diagnosis of ORC (if any) was required to be classified as exposed.

### Mortality outcomes

The primary outcomes were all-cause mortality and ORC-specific mortality. Time to event was calculated in months from the date of BrCa, CRC, ECa, or OCa diagnosis to either death or the end of the follow-up (right-censored), whichever comes first. Death from any cause was considered the event for the all-cause mortality analysis. A competing risk analysis evaluated ORC-specific mortality, with death from other causes as a competing risk. Given that the SEER database only records diagnosis month and year, we designated the first of the corresponding month as the day of diagnosis to handle censoring more effectively.

### Covariates

Study covariates were selected based on *a priori knowledge*^46^. Patient characteristics included age at index date (65–70, 70–75, 75–80, and ≥ 80 years), race and ethnicity (White, Black, Hispanics, and other), Percent of residents living below the poverty, and NCI-Charlson Comorbidity Index (CCI 0, 1, 2, 3 or more). The CCI was calculated six months prior to the index date to assess the comorbidity burden. Clinical indicators identified from Medicare claims using NDC and CPT codes (available upon request), included diabetes, insulin use, hypertension, hyperlipidemia, cardiovascular disease, fatigue, muscle wasting, hypogonadism, pituitary dysfunction, depression, osteoporosis, Cushing syndrome, hypothyroidism, hyperthyroidism, and polycystic ovary syndrome. To minimize ascertainment bias, the covariates were measured at least six months before the index date and the mandatory six-month gap between medication initiation and ORC diagnosis.

### Statistical analysis

Descriptive statistics were used to summarize the baseline characteristics of the study cohort. Categorical variables were presented as frequencies and proportions, while continuous variables were described using means and standard deviations (SDs). Study variables were compared using the Chi-square test or Fisher exact test, as appropriate. Overall survival (OS) probabilities were calculated using the Kaplan-Meier method and compared between the metformin and WLM groups using the log-rank test.

To adjust for potential confounding due to baseline differences across exposure groups, we employed inverse probability of treatment weighting (IPTW) based on propensity scores in a multi-category exposure setting^47^. A propensity score is the estimated probability that an individual receives a particular treatment, given their observed baseline characteristics. By weighting individuals based on these probabilities, IPTW creates a pseudo-population in which the distribution of observed covariates is independent of treatment assignment, thus mimicking some of the properties of a randomization. Our study involved four mutually exclusive exposure groups: neither users (reference group), metformin alone, WLM alone, and dual users. Because traditional propensity score methods are designed for binary exposures, we used a multiple binary comparison approach: we fit three separate binary propensity score models, each comparing one active treatment group to the reference group. Each model was estimated using gradient boosting machines (GBMs) with cross-validation and included all baseline covariates. For each participant, we calculated the inverse of their estimated probability of being in their observed treatment group (inverse propensity score), producing IPTWs. These weights were then averaged across the three models to derive a final stabilized weight for each individual. These stabilized IPTWs were applied in subsequent multivariable models to reduce confounding and estimate adjusted associations between exposure and outcome. We assessed the convergence of the propensity score models and evaluated covariate balance before and after weighting using standardized mean differences (Supplemental Figs. 3–5). The IPTW approach resulted in substantial reduction in covariate imbalances across exposure groups, indicating that the weighting strategy successfully addressed confounding in our analytic sample.

Multivariable Cox proportional hazards models were employed to assess all-cause mortality risk before and after IPTW adjustment. Competing risk analyses were performed using the Fine-Gray (FG) subdistribution hazard models before and after IPTW adjustment to account for the possibility of death from causes other than ORC. The FG model is designed to model the cumulative incidence of events of interest and estimate the marginal probability of an event in the presence of competing events that may prevent the event of interest from occurring. Potential interactions between metformin or WLM and other covariates were evaluated prior to model selection. The proportionality assumption was assessed using log-log survival plots and weighted Schoenfeld residuals. Model goodness-of-fit was evaluated using Cox-Snell and deviance residuals. Values of *P* < 0.05 were considered statistically significant. Statistical modeling was performed using SAS (SAS Institute v.9.4, Cary, NC, USA, RRID: SCR_008567), and result figures were generated using R software (RStudio, v.4.3.1, Boston, MA, USA, RRID: SCR_000432).

## Results

Table 1 summarizes characteristics of 63,907 women aged ≥ 65 years diagnosed with ORC in the SEER-Medicare dataset (2007–2015), including BrCa (*n* = 40,909), CRC (*n* = 23,473), (ECa (*n* = 6,654), and OCa (*n* = 2,736). Most patients (75.9%) had no pre-diagnostic exposure to metformin or WLM; 22.3% used metformin alone, 0.9% used WLM alone, and 0.9% used both. Users of metformin and/or WLM were generally younger, more likely to be White, and had a higher burden of comorbidities including diabetes, hypertension, cardiovascular disease, and various metabolic and endocrine conditions. Characteristics of the study cohort according to the ORC type are presented in Supplemental Table 3. The median follow-up was 43.2 months for the ORC cohort overall, ranging from 22.1 months in OCa to 46.2 months in BrCa. The estimated 5-year OS in the ORC cohort was highest among non-users (79.7%), followed by dual users (75.7%), WLM users (69.9%), and metformin users (59.7%) (log-rank *p* < 0.0001) (Supplemental Table 4).

**Table 1:** Characteristics of the study cohort, *n =* 63,907.

Characteristics	Neither users	Metformin alone	WLM alone	Dual users	p-value
	N ( % )	N ( % )	N ( % )	N ( % )	
	48,486 (75.87)	14,239 (22.28)	605 (0.95)	577 (0.90)	
Incident ORC					<0.0001^a^
Breast cancer	31,871 (65.73)	8,252 (57.95)	401 (66.28)	385 (66.72)	
Colorectal cancer	9,865 (20.35)	3,520 (24.72)	114 (18.84)	109 (18.89)	
Endometrial cancer	4,719 (9.73)	1,809 (12.70)	59 (9.75)	67 (11.61)	
Ovarian cancer	2,031 (4.19)	658 (4.62)	31 (5.12)	16 (2.77)	
ORC Stage^d^					<0.0001^a^
Localized stage	39,945 (82.38)	10,865 (76.30)	491 (81.16)	481 (83.36)	
Advanced stage	8,541 (17.62)	3,374 (23.70)	114 (18.84)	96 (16.64)	
ORC Grade^e^					<0.0001^a^
Low grade	38,130 (78.64)	10,865 (76.30)	453 (74.88)	446 (77.30)	
High grade	10,356 (21.36)	3,374 (23.70)	152 (1.08)	131 (22.70)	
Age at index date					<0.0001^a^
65 – 70	6,547 (13.50)	1,614 (11.34)	124 (20.50)	113 (19.58)	
70 – 75	13,985 (28.84)	4,239 (29.77)	202 (33.39)	239 (41.42)	
75 – 80	12,294 (25.36)	3,793 (26.64)	130 (21.49)	143 (24.78)	
≥ 80	15,660 (32.30)	4,593 (32.26)	149 (24.63)	82 (14.21)	
Race/ethnicity					<0.0001^a^
White	40,537 (83.61)	10,400 (73.04)	>518 (>85.79)	459 (79.55)	
Black	4,186 (8.63)	2,103 (14.77)	54 (8.93)	67 (11.61)	
Hispanic	>797 (>1.63)	535 (3.76)	<11 (<1.65)^c^	22 (3.81)	
Other	2,965 (6.12)	1,201 (8.43)	22 (3.64)	29 (5.03)	
Charlson comorbidity index					<0.0001^a^
0	31,693 (65.37)	2,082 (14.62)	246 (40.66)	95 (16.46)	
1	10,438 (21.53)	6,639 (46.63)	169 (27.93)	223 (38.65)	
2	3,952 (8.15)	3,321 (23.32)	114 (18.84)	146 (25.30)	
3 or more	2,403 (4.96)	2,197 (15.43)	76 (12.56)	113 (19.58)	
Diabetes	5,691 (11.74)	11,802 (82.89)	135 (22.31)	444 (76.95)	<0.0001^a^
Use of insulin	1,115 (2.30)	1,694 (11.90)	41 (6.78)	99 (17.16)	<0.0001^a^
Hypertension	24,917 (51.39)	10,673 (74.96)	398 (65.79)	435 (75.39)	<0.0001^a^
Hyperlipidemia	17,419 (35.93)	7,652 (53.74)	266 (43.97)	316 (54.77)	<0.0001^a^
Cardiovascular disease	20,689 (42.67)	7,544 (52.98)	352 (58.18)	333 (57.71)	<0.0001^a^
Malaise and fatigue	6,390 (13.18)	2,410 (13.18)	170 (16.93)	120 (28.10)	<0.0001^a^
Muscular wasting and atrophy	>422 (>0.85)	144 (1.01)	16 (2.64)	<11 (<1.25)^c^	<0.0001^a^
Hypogonadism^b^	<11 (<0.05)^c^	<11 (<0.05)^c^	0 (0.0)	0 (0.0)	0.3715
Anterior pituitary dysfunction^b^	30 (0.06)	13 (0.09)	0 (0.0)	0 (0.0)	0.675
Depression disorder	1,970 (4.06)	792 (5.56)	71 (11.74)	55 (9.53)	<0.0001^a^
Osteoporosis	5,764 (11.89)	1,266 (8.89)	81 (13.39)	54 (9.36)	<0.0001^a^
Cushing’s syndrome^b^	<11 (<0.05)^c^	<11 (<0.08)^c^	<11 (<0.25)^c^	0 (0.00)	0.0392
Hypothyroidism	7,875 (16.24)	2,706 (19.00)	174 (28.76)	127 (22.01)	<0.0001^a^
Hyperthyroidism	>511 (>1.00)	175 (1.23)	14 (2.31)	<11 (<1.10)^c^	0.0108^a^
Polycystic ovary syndrome^b^	<11 (<0.05)^c^	<11 (<0.05)^c^	0 (0.00)	0 (0.00)	0.2543
Percent of residents living below poverty, mean (SD)	10.84 (8.42)	12.96 (9.37)	11.02 (8.42)	12.87 (8.94)	<0.0001^a^

ORC, obesity-related cancer; SD, standard deviation; WLM, weight loss medication.

^a^Denote Chi-square statistical significance at the *p*-value < 0.05 level.

^b^*p*-value for fisher exact test.

^c^SEER-Medicare data presentation guideline has been followed and all counts less than 11 have been suppressed.

^d^Advanced stage ORC indicated AJCC stage III & IV definition while localized stage ORC indicated stage I & stage II.

^e^High grade ORC indicated G3 (poorly differentiated) and G4 (undifferentiated). Low grade indicated G1(well differentiated) and G2 (moderately differentiated).

**Fig. 2:**
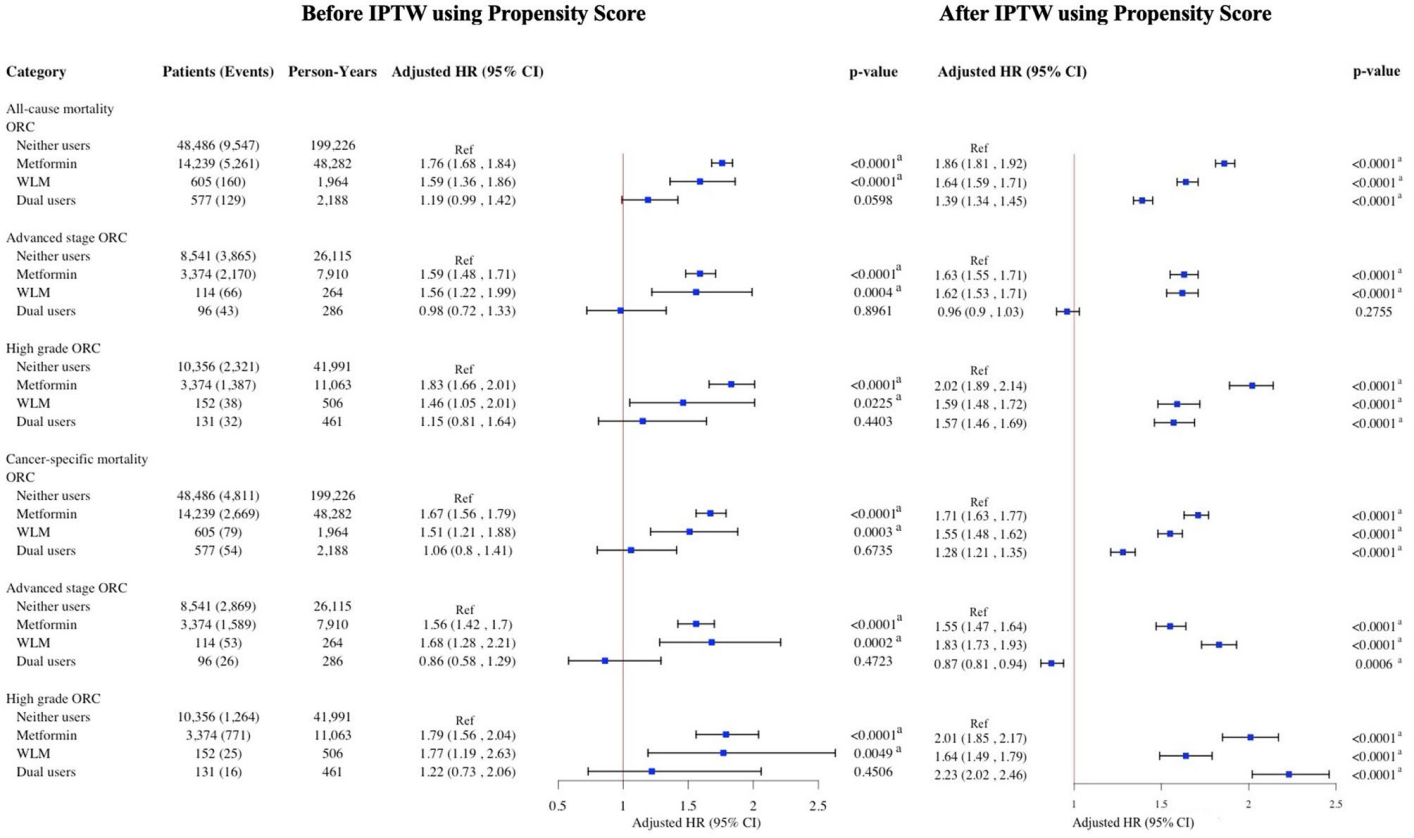
HRs and 95% CIs for the association of pre-diagnostic use of metformin and WLM with all-cause and cancer-specific mortality among women 65 + years old with ORC. aHR, adjusted hazard ratio; CI, confidence interval; ORC, obesity-related cancers; WLM, weight loss medication. Multivariable Cox regressions (all-cause mortality) and Fine-Gray competing risks (cancer-specific mortality) models are adjusted for age at prescription, race/ethnicity, cancer stage, cancer grade, Charlson comorbidity index, diabetes, use of insulin, hypertension, hyperlipidemia, cardiovascular disease, malaise and fatigue, muscular wasting and atrophy, hypogonadism, anterior pituitary dysfunction, depression, osteoporosis, Cushing syndrome, hypothyroidism, hyperthyroidism, polycystic ovary syndrome, poverty rate. ^a^Denotes statistical significance at the P-value < 0.05 level.

**Fig. 3:**
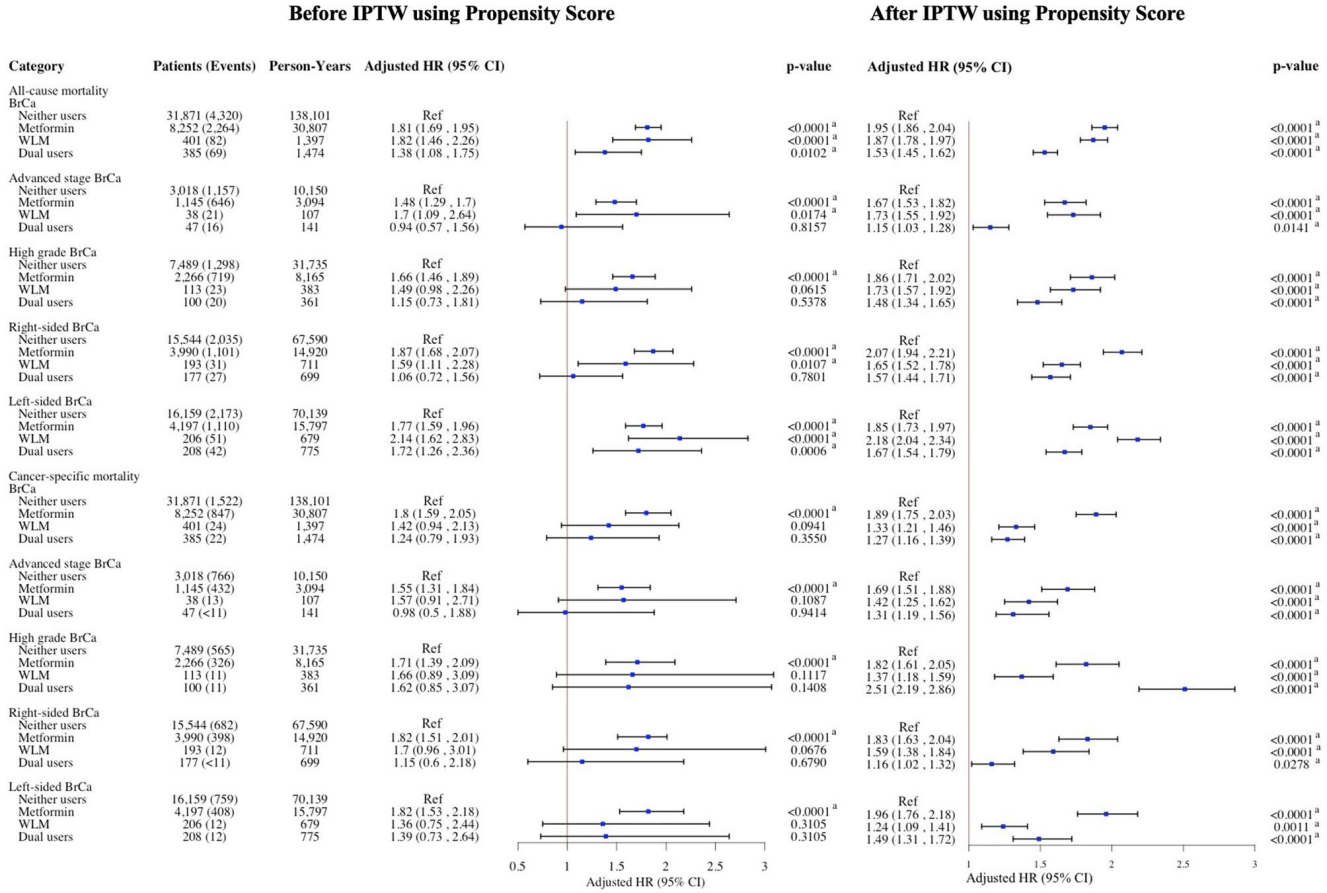
HRs and 95% CIs for the association of pre-diagnostic use of metformin and WLM with all-cause and cancer-specific mortality among women 65 + years old with BrCa. aHR, adjusted hazard ratio; BrCa, breast cancer; CI, confidence interval; WLM, weight loss medication. Multivariable Cox regressions (all-cause mortality) and Fine-Gray competing risks (cancer-specific mortality) models are adjusted for age at prescription, race/ethnicity, cancer stage, cancer grade, Charlson comorbidity index, diabetes, use of insulin, hypertension, hyperlipidemia, cardiovascular disease, malaise and fatigue, muscular wasting and atrophy, hypogonadism, anterior pituitary dysfunction, depression, osteoporosis, Cushing syndrome, hypothyroidism, hyperthyroidism, polycystic ovary syndrome, poverty rate. ^a^Denotes statistical significance at the P-value < 0.05 level. ^b^SEER-Medicare data presentation guideline has been followed and all counts less than 11 have been suppressed.

**Fig. 4:**
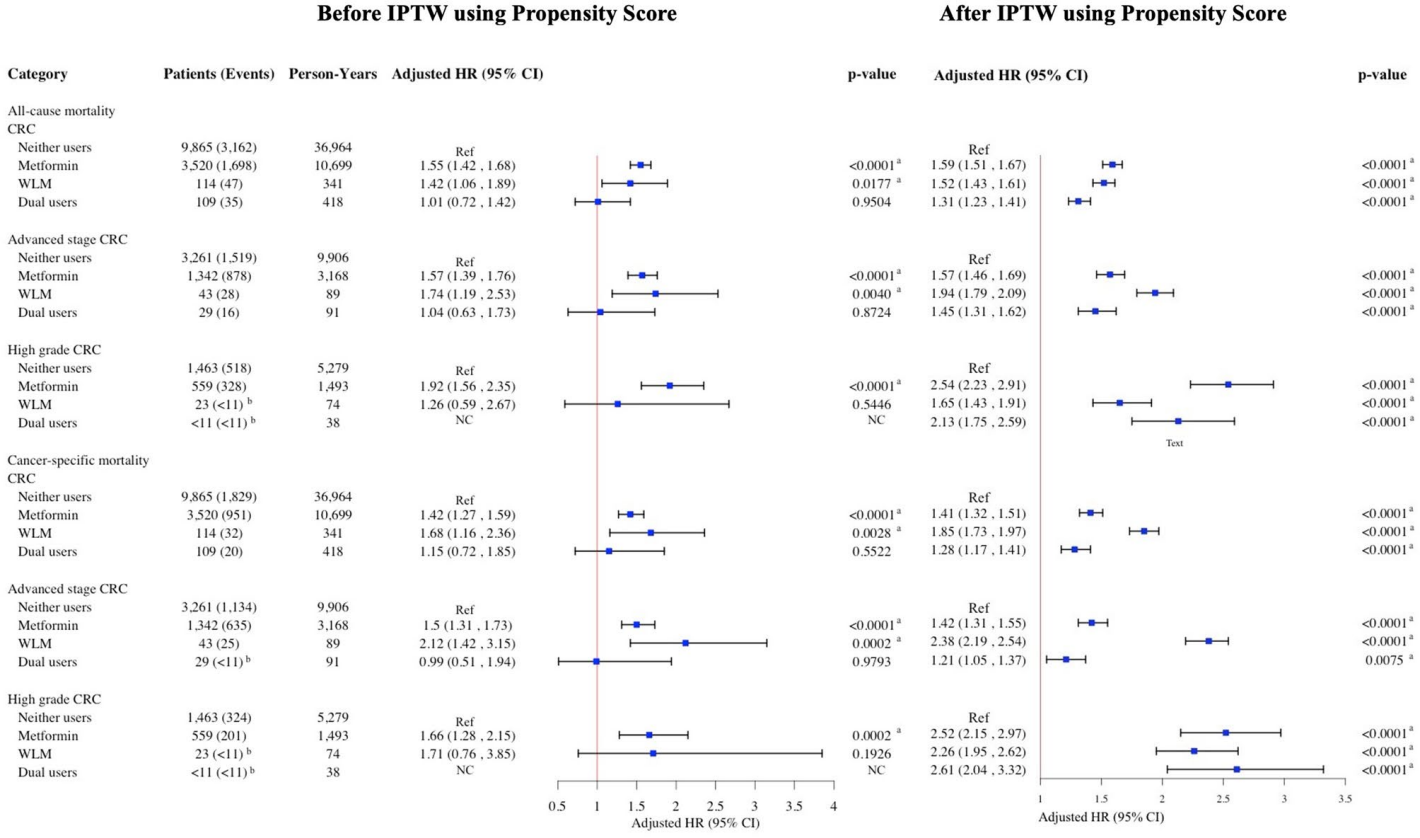
HRs and 95% CIs for the association of pre-diagnostic use of metformin and WLM with all-cause and cancer-specific mortality among women 65 + years old with CRC. aHR, adjusted hazard ratio; CRC, colorectal cancer; CI, confidence interval; NC, not calculated; WLM, weight loss medication. Multivariable Cox regressions (all-cause mortality) and Fine-Gray competing risks (cancer-specific mortality) models are adjusted for age at prescription, race/ethnicity, cancer stage, cancer grade, Charlson comorbidity index, diabetes, use of insulin, hypertension, hyperlipidemia, cardiovascular disease, malaise and fatigue, muscular wasting and atrophy, hypogonadism, anterior pituitary dysfunction, depression, osteoporosis, Cushing syndrome, hypothyroidism, hyperthyroidism, polycystic ovary syndrome, poverty rate. ^a^Denotes statistical significance at the P-value < 0.05 level. ^b^SEER-Medicare data presentation guideline has been followed and all counts less than 11 have been suppressed.

**Fig. 5:**
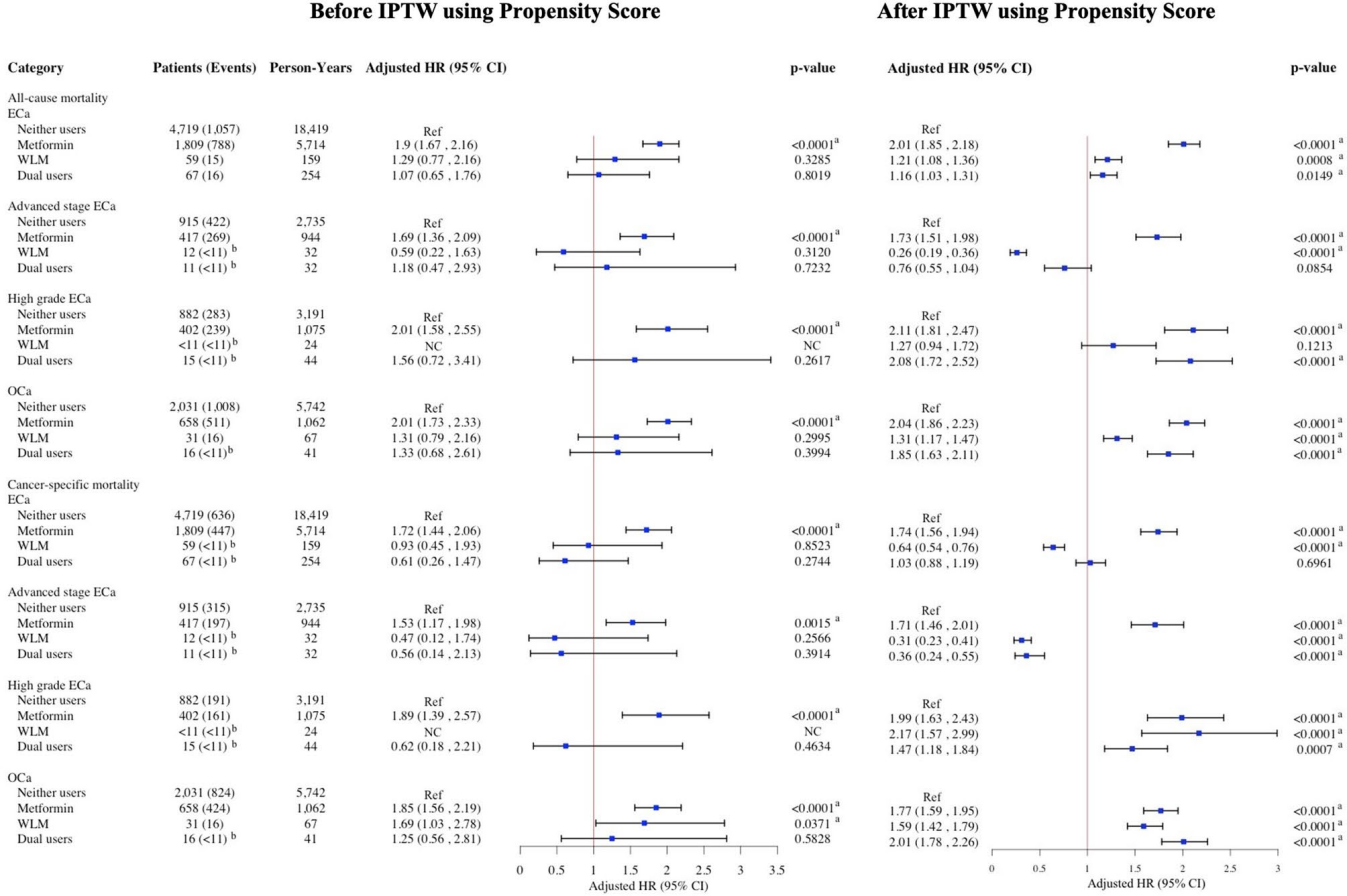
HRs and 95% CIs for the association of pre-diagnostic use of metformin and WLM with all-cause and cancer-specific mortality among women 65 + years old with ECa and OCa. Abbreviations: aHR, adjusted hazard ratio; CI, confidence interval; ECa, endometrial cancer; NC, not calculated; OCa, ovarian cancer; WLM, weight loss medication. Multivariable Cox regressions (all-cause mortality) and Fine-Gray competing risks (cancer-specific mortality) models are adjusted for age at prescription, race/ethnicity, cancer stage, cancer grade, Charlson comorbidity index, diabetes, use of insulin, hypertension, hyperlipidemia, cardiovascular disease, malaise and fatigue, muscular wasting and atrophy, hypogonadism, anterior pituitary dysfunction, depression, osteoporosis, Cushing syndrome, hypothyroidism, hyperthyroidism, polycystic ovary syndrome, poverty rate. ^a^Denotes statistical significance at the P-value < 0.05 level. ^b^SEER-Medicare data presentation guideline has been followed and all counts less than 11 have been suppressed.

After inverse probability of treatment weighting using propensity scores, pre-diagnostic metformin use was associated with increased risks of all-cause and cancer-specific mortality across all cancer types. In the overall ORC cohort, metformin use was linked to an 86% increase in all-cause mortality and a 71% increase in ORC-specific mortality; WLM use was associated with a 64% and 55% increase, respectively, and dual use with a 39% and 28% increase (Fig. 2). In BrCa, metformin was associated with a 95% increase in all-cause mortality and 89% in BrCa-specific mortality, WLM with 87% and 33%, and dual use with 53% and 27% (Fig. 3). Among CRC patients, respective increases in all-cause and cancer-specific mortality were 59% and 41% for metformin, 52% and 85% for WLM, and 31% and 28% for dual use (Fig. 4). In ECa, metformin use was associated with a 101% increase in all-cause mortality and 74% in ECa-specific mortality, while WLM was linked to a 21% increase in all-cause mortality and a 36% reduction in ECa-specific mortality; dual use was associated with a 16% increase in all-cause mortality without a clear effect on cancer-specific mortality (Fig. 5). For OCa, metformin, WLM, and dual use were associated with 104%, 31%, and 85% increases in all-cause mortality, respectively, with similar trends observed for cancer-specific mortality (Fig. 5). These associations were consistent across subgroups of patients with advanced stage or high grade cancer (Figs. 2, 3, 4 and 5). Subgroups results are detailed in the supplemental materials.

In the sensitivity analysis (Supplementary Tables 5, 6), we separately assessed the association between metformin and WLM with all-cause and cancer-specific mortality in older women with ORC, distinguishing between women with and without diabetes. Overall, while the direction and magnitude of these associations were largely consistent with the primary analysis, diabetes status appeared to modulate the impact of metformin and WLM on mortality risk in older women with ORC. Interestingly, in the non-diabetic subgroup, metformin users generally had a higher mortality risk, while in the diabetic subgroup, WLM users had a greater risk compared to the other groups.

## Discussion

This large population-based retrospective cohort study set out to examine the association between pre-diagnostic use of metformin and WLM with the risks of all-cause and cancer-specific mortality among older women diagnosed with ORC, which included BrCa, CRC, ECa, and OCa. We observed an increased risk of all-cause and ORC-specific mortality associated with metformin alone, WLM alone, and the combined use of both. These findings persisted in ORC subgroup analyses when stratified by cancer type (BrCa, CRC, and OCa), stage, and grade.

Unexpectedly, our findings consistently demonstrated a significantly increased risk of all-cause and cancer-specific mortality associated with metformin use across all ORC types studied. This contradicts the prevailing hypothesis of metformin’s survival advantage, particularly in the context of diabetes and cancer^13–17^. In total ORC, metformin use was associated with increased risks of all-cause and ORC-specific mortality, with these associations persisting in advanced and high-grade disease. Similar findings were observed in BrCa and its subgroups (advanced, high grade, right-sided, left-sided), where metformin use was associated with increased risks of all-cause and BrCa-specific mortality. While observational studies have suggested a potential survival benefit for metformin in BrCa^48–51^, this was not confirmed in RCTs^52–54^. Notably, a previous study reported increased risks of overall and BrCa-specific mortality following metformin discontinuation^55^. The observed associations between metformin use and increased mortality in CRC, ECa, and OCa diverge from the majority of the literature^14,15,56,57^. However, two studies have reported increased cancer-specific mortality among metformin users^18,19^.

Our study identified associations between WLM use and increased risks of all-cause and ORC-specific mortality, with these associations persisting in advanced and high grade ORC. WLM use was associated with increased all-cause and BrCa-specific mortality in advanced stage, high grade, right-sided, and left-sided BrCa. In CRC (advanced stage and high grade), WLM use was associated with all-cause and CRC-specific mortality. Likewise, WLM use was associated with all-cause and OCa-specific mortality. In ECa, WLM showed heterogenous results where the risk of all-cause mortality increased in total ECa and decreased in advanced stage ECa. In addition, the risk of ECa-specific mortality was decreased in total ECa and advanced stage subgroup but increased in high grade subgroup. A recent meta-analysis reported a decreased all-cause mortality with liraglutide use^58^, but its target population differed from our study cohort, as the average age reported was significantly younger (59.4 years) compared to our study’s average age of 76.8 years. Likewise, studies investigating orlistat and phentermine found no increased risk of all-cause mortality^59–61^ yet these studies also involved populations that were considerably younger than ours.

In our study, the combined use of metformin and WLM was generally associated with an increased risk of all-cause and cancer-specific mortality across the overall ORC cohort and among those with high-grade disease. Interestingly, in advanced-stage ORC, this pattern appeared to differ, with a potential reduction in ORC-specific mortality observed among dual users. Similar patterns were noted within individual cancer types (BrCa, CRC, ECa, and OCa) where dual use was generally linked to increased mortality, except in advanced-stage ECa, where a lower risk of ECa-specific mortality was observed. These findings are exploratory and should be interpreted with caution, particularly in light of the limited number of dual users, which may impact the stability of the estimates. To our knowledge, the combined use of metformin and WLM association with mortality in patients with ORC has not been previously studied. The paucity of research on combined use of metformin and WLM and mortality outcomes in older women with ORCs limits the direct comparison of our findings with existing literature.

The observed increased mortality risk associated with metformin is particularly surprising given the substantial body of literature supporting its potential survival benefits^11–13^. The mechanisms by which metformin and WLM influence cancer mortality are likely complex, involving both direct and indirect effects. The former suggests that the chemical properties of these medications can potentially inhibit cancer cell proliferation^30^, while the latter posits that weight loss can modify the adipose tissue microenvironment with concomitant systemic endocrine alterations in ways that suppress tumor development and progression^31^. Our findings align with a minority of studies reporting adverse cancer outcomes linked to metformin use^14,15^, they suggest a poorer cancer prognosis than is typically reported in the majority of the literature. One possible biological mechanism for poor prognosis associated with metformin and WLM is the ability of these medications to modulate the immune system. Metformin can exert immunosuppressive effects by reducing pro-inflammatory cytokine production and promoting regulatory T cell differentiation. Additionally, metformin has been shown to modulate immune responses by reducing reactive oxygen species (ROS) levels in immune cells, which may weaken the immune response and increase susceptibility to infections and complications from cancer treatments^32,33^. WLM affect lipid metabolism and have less direct evidence regarding their impact on the immune system. However, there is limited evidence suggesting that WLM have significant immunomodulatory effects beyond their metabolic actions^34^.

The observed association between pre-diagnostic use of metformin or WLM and increased mortality risk may be driven by underlying clinical and methodological factors. Individuals prescribed metformin or WLM often have comorbid conditions that independently confer elevated mortality risk. Although IPTW using propensity scores was employed to balance observable baseline characteristics, this approach does not account for unmeasured confounding, including factors such as diabetes duration, glycemic control, body mass index (BMI), functional status, or frailty. These unmeasured variables may contribute substantially to the increased mortality risk observed in the exposed groups.

Additionally, the discrepancy between our findings and prior studies reporting favorable survival outcomes with metformin use may reflect differences in model specification and covariate adjustment. Our analysis did not account for cancer treatment modalities, such as surgery, chemotherapy, radiotherapy, or hormonal therapy, which are strong prognostic determinants and may have introduced residual confounding or effect modification. For example, if metformin users were less likely to receive aggressive or guideline-concordant treatment due to underlying comorbidities or frailty, the observed increase in mortality may partially reflect differences in treatment intensity rather than a direct adverse effect of the medication itself. In contrast, if non-users were more likely to receive curative treatment, this could further widen survival differences. Furthermore, the potential for pharmacologic interaction between metformin or WLM and cancer-directed therapies remains unexplored in this context. Insulin resistance and metabolic dysregulation may impair response to chemotherapy, while WLM use may alter drug metabolism, nutrient absorption, or immune function, potentially affecting treatment efficacy. These complex and unmeasured interrelationships underscore the need for cautious interpretation of our findings and reinforce the importance of future studies that incorporate detailed treatment data to disentangle the effects of metabolic medications from underlying clinical trajectories and therapeutic exposures.

The complicated relationship between obesity, weight loss and management, and cancer mortality has been a subject of ongoing research. One possible explanation for the observed association between metformin and WLM use with increased mortality risk in ORC is the obesity paradox, wherein weight loss might paradoxically lead to a poorer prognosis^41–44^. In the context of cancer, the link between the obesity paradox and risk of mortality remains controversial due to the concomitant weight loss and cachexia, further complicated by the timing and rate of weight loss. However, this potential link in our study remains speculative due to insufficient evidence. Unfortunately, our study lacked BMI data, precluding a direct assessment of weight changes among ORC patients. Therefore, we cannot definitively establish an association between weight reduction due to metformin or WLM use and risk of mortality. Hence, speculation on this topic is therefore unwarranted. A prospective study of older women found that overweight women who experienced minimal weight loss (< 5%) demonstrated a significantly lower risk of mortality at both 12 and 48 months compared to normal-weight women with similar weight loss. Women with obesity and minimal weight loss also exhibited lower mortality risk at 48 months. Interestingly, this survival benefit was more pronounced in overweight women with advanced cancer^31^. These findings support the concept of the obesity paradox, particularly among older women with cancer mortality, as it was previously shown by Flegal et al. reporting that overweight reduced the risk of mortality^62^.

Moreover, the observed association between pre-diagnostic metformin and WLM use and increased mortality risk in older women with ORC may be partially attributable to the prescription practices during the period when the study data was collected (2007–2015). Over the past two decades, the adoption of WLM and metformin for obesity management by clinicians has been characterized by cautious and limited utilization. Clinicians generally perceived metformin as well-tolerated and a safe option for managing diabetes. While metformin, although not FDA-approved for obesity, was occasionally prescribed off-label for weight management, particularly for patients with more severe obesity or multiple comorbidities^11,63^, which could diminish potential metformin benefits and complicate their overall prognosis and treatment response for those with ORC. The U.S. Preventive Services Task Force acknowledged modest weight loss and improved physiological outcomes with metformin when combined with behavioral interventions^64^. However, its primary role remained glycemic control. The conservative approach to pharmacotherapy for obesity in the past two decades resulted in limited long-term efficacy studies. The WLM was often prescribed as adjuncts to lifestyle changes, with a strong preference for established agents like phentermine, accounting for over 75% of all WLM prescriptions^65^. A study analyzing data from 1999 to 2010 revealed a remarkably low prescription rate for WLM, with only 2% of obesity-related visits mentioning such medications^66^. While newer WLM groups were introduced, their adoption rates exhibited a linear rather than exponential trajectory, suggesting systemic barriers to their widespread use^67^. Furthermore, the utilization of medications like orlistat and sibutramine significantly declined over time, likely attributable to concerns regarding safety and limited long-term efficacy^68^.

Our findings suggest a concerning association between pre-diagnostic use of metformin and WLM with increased mortality risk in older women with ORCs. This necessitates further investigation through several key avenues. Firstly, large-scale epidemiological studies should examine the long-term effects of pre-diagnostic metformin and WLM use on overall survival and cancer-specific outcomes, considering time- and dose-response. Secondly, rigorously designed RCTs are crucial to establish a causal relationship between these medications and mortality outcomes, while simultaneously evaluating their safety and efficacy within the ORC population. Finally, laboratory-based studies should explore the underlying biological mechanisms of these associations. These studies should investigate how metformin and WLM influence tumor biology, including their impact on metabolic alterations, inflammation, and insulin resistance. Through these concerted research efforts, we can gain a deeper understanding of the complex interplay between these medications, cancer progression, and mortality in this vulnerable population. This knowledge is critical for developing more safe, informed, and personalized treatment strategies for older women with ORC.

This study has strengths. The SEER-Medicare large sample size attenuates the random error and confers adequate statistical power to detect subtle effects. We managed immortal bias effectively by defining appropriate time windows for metformin and WLM exposure and limiting our analysis to patients diagnosed with ORC after the follow-up period began and who survived at least six months after ORC diagnosis. Nonetheless, there are several limitations to consider. The retrospective nature of the study may introduce selection bias. While the focus of this study is on pre-diagnostic exposure, it does not account for the duration, dosage, or adherence to metformin or WLM. The available data did not provide sufficient information on these aspects, and as such, variability in patients’ exposure to these medications could not be assessed. Additionally, we did not assess the use of other diabetes medications, as this was outside the scope of the study and not feasible given the complexity of individual treatment regimens and limitations of the SEER-Medicare data. As a result, individuals using alternative diabetes therapies may have been classified in the ‘neither’ group or another exposure category, introducing heterogeneity into the comparison groups. While this may have confounded the observed associations, adjusting for every possible medication was neither technically practical nor methodologically appropriate, as it could introduce overadjustment bias, multicollinearity, and reduced model interpretability. The six-month pre-index period might not fully capture the patient’s medical history, and comorbidities may have existed prior to this timeframe. The relatively short follow-up period, especially for OCa, may limit our ability to estimate long-term mortality risk accurately. Despite adjusting for potential confounders in multivariable models, the impact of unmeasured factors such as genetics, lifestyle habits (physical activity, smoking, and alcohol consumption), and residual confounding cannot be completely ruled out. The SEER-Medicare indicates that laboratory results or other environmental, and nutritional/lifestyle factors are incompletely reported with low sensitivity. This includes data for BMI (≥ 30 kg/m2) to define obesity, which is a risk factor for mortality. The underreporting and inaccurate coding of BMI limit the completeness and reliability of analyses that rely on BMI. While SEER-Medicare does not explicitly imply that BMI should not be used, it labeled BMI as problematic and could lead to misclassification bias or inaccurate adjustments^69^. However, in a previous investigation for sensitivity analyses we adjusted our multivariable models by including obesity using diagnostic ICD-10 codes (e.g., E66.01, 0.2, 0.3, and Z68/35–39), and the results remained nearly identical to the ones without obesity due to its low sensitivity in SEER-Medicare^70^. Furthermore, in the present study we adjusted for strong factors associated with obesity such as diabetes, hypertension, CCI comorbidity score, hyperlipidemia, and use of insulin that it is possible that some levels of obesity were captured and adjusted for^46^, yet residual confounding may remain. Our dataset lacks information on important prognostic markers in BrCa, including hormone receptor (HR) and HER2 (human epidermal growth factor receptor 2) status. This limitation prevented us from analyzing HR/HER2 subgroups in this study. We also did not account for cancer treatment modality due to data reliability issues such as incomplete data, biases related to unmeasured factors influencing the decision to receive treatment, and difficulties in interpreting sequence data variables^71^. However, cancer treatment modality is likely to modify the observed effects rather than simply confounding them. Our study speaks only for women aged 65 and older diagnosed with ORC and cannot be generalized to other patient populations. Medicare claims data is susceptible to errors and omissions, leading to selection and measurement biases that may significantly impact the reproducibility and comparability of studies. The earliest available Part D in SEER-Medicare data was from 2007; any use of metformin and WLM prior to that was not accounted for. Given these aforementioned limitations, conclusions should be drawn with caution.

## Conclusion

In summary, this population-based cohort study employed multivariable Cox regression and competing-risks models with IPTW-PS to investigate the association between pre-diagnostic use of metformin and WLM with mortality outcomes in older women with ORC, including BrCa, CRC, ECa, and OCa. We observed an increased risk of all-cause and ORC-specific mortality associated with independent and joint use of metformin and WLM across various ORC subtypes. Pre-diagnostic metformin and WLM use, alone and in combination, was associated with an increased risk of all-cause mortality in BrCa (advanced stage, high grade, right-sided, left-sided), CRC (advanced stage and high grade), and OCa. In ECa, WLM showed heterogenous results where the risk of all-cause mortality increased in total ECa and decreased in advanced stage ECa. Additionally, the risk of ECa-specific mortality was decreased in overall ECa and advanced stage subgroup but increased in high grade subgroup. While the evidence regarding metformin’s impact on mortality in ovarian cancer remains inconclusive, our findings challenge the prevailing notion of its potential protective effect. The limited research on WLM use and mortality outcomes in ORC hinders direct comparison of our results. However, given the substantial burden of ORC and increasing interest in weight loss treatments, further investigation into the safety and prognostic implications of WLM is warranted. Large-scale prospective studies with extended follow-up periods are needed to solidify these findings and inform clinical practice.

## Implications for cancer survivors

Our findings may stimulate research into alternative weight management strategies for older women with ORCs that are effective and do not carry the same potential risks.

## Electronic supplementary material

Below is the link to the electronic supplementary material.

Supplementary Material 1

Supplementary Material 2

## Data Availability

The SEER-Medicare datasets used to conduct this study are available upon approval of a research protocol by the National Cancer Institute. Instructions for obtaining these data are available at https://healthcaredelivery.cancer.gov/seermedicare/obtain/.

## Abbreviations

aHR: Adjusted hazard ratio
BMI: Body mass index
BrCa: Breast cancer
CCI: Charlson comorbidity index
CI: Confidence interval
CRC: Colorectal cancer
CPT: Current procedural terminology
ECa: Endometrial cancer
FG: Fine-Gray
HR: Hormone receptor
HER2: Human epidermal growth factor receptor 2
IPTW: Inverse probability of treatment weighting
NDC: National Drug Codes
Oca: Ovarian cancer
OS: Overall survival
ORCs: Obesity-related cancers
PS: Propensity score
RCT: Randomized controlled trials
ROS: Reactive oxygen species
SEER: Surveillance, Epidemiology, and End Results
WLM: Weight loss medication
