# Supplementary material for: Metformin and weight loss medication impact on survival outcomes in older women with obesity-related cancers
Source: Sci Rep. 2025 Jul 1;15:21828. doi: 10.1038/s41598-025-09393-1 (PMC12218299; doi:10.1038/s41598-025-09393-1)
Supplement: Supplementary file 1 — Supplementary Material 1 [file 41598_2025_9393_MOESM1_ESM.pdf]

Supplemental Materials

Supplemental Results

Supplemental Table 3 shows estimated 5-year OS rates. The median follow-up duration varied across the cohorts: 43.2 months for ORC, 46.2 months for BrCa, 37.2 months for CRC, 39.2 months for ECa, and 22.1 months for OCa. Additionally, the follow-up duration differed between the groups based on the use of metformin and WLM. In the ORC cohort, the estimated 5-year OS rate was 79.7% for those who did not use metformin or WLM, 59.7% for metformin users, 69.9% for WLM users, and 75.7% for dual users. These OS rates differed significantly (log-rank test $p < 0.0001$).

Figure 2 displays the results of multivariable Cox proportional hazards and competing risk models for the effect of pre-diagnostic use of metformin and WLM on all-cause and cancer-specific mortality among patients with ORCs. After IPTW with PS, compared to neither metformin or WLM use, metformin use was associated with an 86% increased risk of all-cause mortality and a 71% increased risk of ORC-specific mortality. Similarly, WLM use was associated with a 64% increased risk of all-cause mortality and a 55% increased risk of ORC-specific mortality. Dual use of metformin and WLM was associated a 39% increased risk of all-cause mortality and a 28% increased risk of ORC-specific mortality. These associations were consistent across subgroups of patients with advanced stage or high grade ORC except that dual use was associated 13% decreased risk of ORC-specific mortality in advanced stage ORC.

Figure 3 illustrates the results of multivariable Cox proportional hazards and competing risk models for the effect of pre-diagnostic use of metformin and WLM on all-cause and cancer-specific mortality among patients with BrCa. After IPTW with PS, metformin use, as opposed to neither metformin or WLM use, was associated with a 95% increased risk of all-cause mortality

and an 89% increased risk of BrCa-specific mortality. Likewise, WLM use was associated with an 87% increased risk of all-cause mortality and a 33% increased risk of BrCa-specific mortality. Combined use of metformin and WLM was associated with a 53% increased risk of all-cause mortality and a 27% increased risk of BrCa-specific mortality. These association persisted across BrCa subgroups including advanced stage, high grade, right-sided, and left-sided BrCa.

Figure 4 shows the result of multivariable Cox proportional hazards and competing risk models for the effect of pre-diagnostic use of metformin and WLM on all-cause and cancer-specific mortality among patients with CRC diagnosis. After IPTW with PS, compared to neither metformin or WLM use, metformin use was associated with a 59% increased risk of all-cause mortality and a 41% increased risk of CRC-specific mortality. Similarly, WLM use was associated with a 52% increased risk of all-cause mortality and an 85% increased risk of CRC-specific mortality. Dual use of metformin and WLM was associated with a 31% increased risk of all-cause mortality and a 28% increased risk of CRC-specific mortality. These associations were maintained in advanced stage and high grade CRC subgroups.

Figure 5 presents the result of multivariable Cox proportional hazards and competing risk models for the independent and joint effect of pre-diagnostic use of metformin and WLM on all-cause and cancer-specific mortality among patients with ECa and OCa diagnosis. After IPTW with PS, metformin use, as opposed to neither metformin or WLM use, was associated with a 101% increased risk of all-cause mortality and a 74% increased risk of ECa-specific mortality, with a similar association in advanced stage and high grade ECa. WLM use was associated with a 21% increased risk of all-cause mortality, while it was associated with a 36% decreased risk of ECa-specific mortality. In advanced stage ECa, WLM was associated with a 74% and 65% decreased risk of all-cause and ECa-specific mortality, respectively. In high grade ECa, WLM

was associated with a two-fold increased risk of ECa-specific mortality. Combined use of metformin and WLM was associated with a 16% increased risk of all-cause mortality, with similar associations observed in high grade ECa subgroup. In advanced stage ECa, Combined use of metformin and WLM was associated a 64% decreased risk of ECa-specific mortality. In the OCa, metformin, WLM and dual use were associated an increased risk of all-cause mortality - 104%, 31%, and 85%, respectively. These associations were also consistent for the risk of OCa-specific mortality.

In the sensitivity analysis (Supplementary Tables 5 – 6) evaluating cancer-specific mortality after IPTW, the associations between metformin, WLM, and mortality were assessed separately for non-diabetic and diabetic women with ORC. Among the non-diabetic subgroup, metformin use (HR: 1.89, 95% CI: 1.79–1.99), WLM use (HR: 1.29, 95% CI: 1.22–1.38), and dual use (HR: 1.50, 95% CI: 1.40–1.60) were all significantly associated with increased all-cause mortality (all $p < 0.0001$). In contrast, among the diabetic subgroup, metformin use (HR: 1.51, 95% CI: 1.41–1.61), WLM use (HR: 2.45, 95% CI: 2.24–2.68), and dual use (HR: 1.14, 95% CI: 1.04–1.25) also showed significant associations with increased all-cause mortality (all $p < 0.0001$, except for dual use with $p = 0.0045$). In the advanced stage ORC cohort, metformin (HR: 1.87, 95% CI: 1.75–1.99) and WLM (HR: 1.55, 95% CI: 1.45–1.66) remained significantly associated with higher all-cause mortality in the non-diabetic subgroup (both p < 0.0001), while metformin (HR: 1.38, 95% CI: 1.26–1.51) and WLM (HR: 3.28, 95% CI: 2.91–3.69) were significant in the diabetic subgroup (both $p < 0.0001$). However, dual use was associated with reduced all-cause mortality in non-diabetics subgroup (HR: 0.75, 95% CI: 0.66–0.84, $p < 0.0001$), while in diabetics subgroup, the association was not significant (HR: 1.10, 95% CI: 0.98–1.24, $p = 0.1045$). In the high grade ORC cohort, metformin (HR: 2.12, 95% CI: 1.92–

2.35), WLM (HR: 1.51, 95% CI: 1.36–1.67), and dual use (HR: 3.94, 95% CI: 3.50–4.44) were all significantly associated with increased mortality in non-diabetics subgroup (all $p < 0.0001$), while in diabetics subgroup, metformin (HR: 1.59, 95% CI: 1.39–1.82), WLM (HR: 2.27, 95% CI: 1.86–2.78), and dual use (HR: 1.22, 95% CI: 1.03–1.44) had significant associations with mortality (all $p < 0.0001$, except for dual use with $p = 0.0221$). Overall, while the direction and magnitude of associations were consistent across groups, diabetes status appeared to modulate the impact of treatment strategies on mortality risk.

Supplemental Table 1: Cancer site, histology and screening codes.

Cancer	Site codes (ICD-O-3)	Histology codes (ICD-O-3)	Screening CPT codes
BrCa	C50.0–C50.9	8500–8543	G0202, G0204, G0206, 76092, 76090, 76091
CRC	C18.0–C20.9	8140–8147, 8210–8211, 8220–8221, 8260–8263, 8480–8481, 8490	45378, 45379, 45380, 45381, 45382, 45388, 45384, 45385, 45386, 45389, 45391, 45392, 45390, 45393, 45398, G0105, G0121
ECa	C54.0–C55.9	8140, 8262, 8323, 8380–8384, 8480–8482, 8560	58100, 58120
OCa	C56.9	8440–8469	76830, 76856, 76857

Abbreviations: BrCa, breast cancer; CRC, colorectal cancer; EC, endometrial cancer; OCa, ovarian cancer;

Supplemental Table 2: Metformin and weight loss medication National Drug Codes (NDC) and Current Procedural Terminology (CPT) codes.

*The Excel file “Supplemental Table 2”.

Supplemental Table 3. Characteristics of the study cohort according to the obesity-related cancer type, n = 63,907.

**Characteristics**	**BrCa**<br>**N (%)**<br>40,909 (64.01)	**CRC**<br>**N (%)**<br>13,608 (21.29)	**ECa**<br>**N (%)**<br>6,654 (10.41)	**OCa**<br>**N (%)**<br>2,736 (4.28)	***p*-value**
Age at index date					<0.0001[a]
• 65 – 70	5,795 (14.17)	1,205 (8.86)	1,099 (16.51)	299 (10.93)	
• 70 – 75	12,607 (30.82)	3,019 (22.19)	2,249 (33.80)	790 (28.87)	
• 75 – 80	10,702 (26.16)	3,309 (24.32)	1,655 (24.87)	694 (25.37)	
• ≥ 80	11,805 (28.86)	6,075 (44.64)	1,651 (24.81)	953 (34.83)	
ORC Stage[d]					<0.0001[a]
• Localized stage	36,661 (89,62)	8,933 (65.65)	5,299 (79.64)	889 (32.49)	
• Advanced stage	4,248 (10.38)	4,675 (34.35)	1,355 (20.36)	1,847 (67.51)	
ORC Grade[e]					<0.0001[a]
• Low grade	30,941 (75.63)	11,553 (84.90)	5,347 (80.36)	2,053 (75.04)	
• High grade	9,968 (24.37)	2,055 (15.10)	1,307 (19.64)	683 (24.96)	
Medication					<0.0001[a]
• Neither users	31.871 (77.91)	9,865 (72.49)	4,719 (70.92)	2,031 (74.23)	
• Metformin alone	8,252 (20.17)	3,520 (25.87)	1,809 (27.19)	658 (24.05)	
• WLM alone	401 (0.98)	114 (0.84)	59 (0.89)	31 (1.13)	
• Dual users	385 (0.94)	109 (0.80)	67 (1.01)	16 (0.58)	
Race/ethnicity					<0.0001[a]
• White	33,545 (82.00)	10.681 (78.49)	5,428 (81.57)	2,261 (82.64)	
• Black	3,990 (9.75)	1,530 (11.24)	681 (10.23)	209 (7.64)	
• Hispanic	813 (1.99)	306 (2.25)	158 (2.37)	88 (3.22)	
• Other	2,561 (6.26)	1,091 (8.02)	387 (5.82)	178 (6.51)	
Charlson comorbidity index					<0.0001[a]
• 0	22,511 (55.03)	6,610 (48.57)	3,541 (53.22)	1,454 (53.14)	
• 1	10,961 (26.79)	3,857 (28.34)	1,914 (28.76)	737 (26.94)	
• 2	4,618 (11.29)	1,831 (13.46)	763 (11.47)	321 (11.73)	
• 3 or more	2,819 (6.89)	1,310 (9.63)	436 (6.55)	224 (8.19)	
Diabetes	10,746 (26.27)	4,352 (31.98)	2,187 (32.87)	787 (28.76)	<0.0001[a]
Use of insulin	1,654 (4.04)	821 (6.03)	354 (5.18)	129 (4.71)	<0.0001[a]
Hypertension	23,138 (56.56)	7,940 (58.35)	3,827 (57.51)	1,518 (55.48)	0.0008[a]
Hyperlipidemia	16,585 (40.54)	5,330 (39.17)	2,643 (39.72)	1,095 (40.02)	0.0352[a]
Cardiovascular disease	18,543 (45.33)	6,313 (46.39)	2,837 (42.64)	1,225 (44.77)	<0.0001[a]
Malaise and fatigue	5,887 (14.39)	1,961 (14.41)	862 (12.95)	380 (13.89)	0.0158[a]
Muscular wasting and atrophy	388 (0.95)	124 (0.91)	48 (0.72)	30 (1.10)	0.2439
Hypogonadism[b]	<11 (<0.05)[c]	<11 (<0.05)[c]	<11 (<0.05)[c]	0 (0.0)	0.8060
Anterior pituitary dysfunction[b]	28 (0.07)	<11 (<0.05)[c]	<11 (<0.05)[c]	0 (0.0)	0.6437

Depression disorder	1,882 (4.60)	609 (4.48)	284 (4.27)	113 (4.13)	0.4542
Osteoporosis	4,650 (11.37)	1,608 (11.82)	557 (8.37)	350 (12.79)	<0.0001[a]
Cushing's syndrome[b]	11 (0.03)	<11 (<0.05)[c]	0 (0.0)	0 (0.0)	0.4987
Hypothyroidism	7,107 (17.37)	2,150 (15.80)	1,112 (16.71)	513 (18.75)	<0.0001[a]
Hyperthyroidism	447 (1.09)	166 (1.22)	59 (0.89)	35 (1.28)	0.1476
Polycystic ovary syndrome[b]	<11 (<0.05)[c]	<11 (<0.05)[c]	0 (0.0)	0 (0.0)	0.6611
Percent of residents living below poverty, mean (SD)	11.10 (8.57)	12.19 (9.02)	11.02 (8.74)	11.36 (8.54)	<0.0001[a]

Abbreviation; BrCa, breast cancer; CRC, colorectal cancer; ECa, endometrial cancer; OCa, ovarian cancer; SD, standard deviation; WLM, weight loss medication.
[a]Denote Chi-square statistical significance at the *p*-value < 0.05 level.
[b]*p*-value for fisher exact test.
[c]SEER-Medicare data presentation guideline has been followed and all counts less than 11 have been suppressed.
[d]Advanced stage ORC indicated AJCC stage III & IV definition while localized stage ORC indicated stage I & stage II.
[e]High grade ORC indicated G3 (poorly differentiated) and G4 (undifferentiated). Low grade indicated G1(well differentiated) and G2 (moderately differentiated).

Supplemental Table 4. Kaplan–Meier estimator for 5-year overall survival rates for women 65+ years old with ORC in SEER-Medicare 2007-2015.

Cohort	Neither users	Metformin alone	WLM[a] alone	Dual users	Log-rank *p*-value
**ORC**					
• Number of patients	48,486	14,239	605	577	
• Median follow-up in months	45.2	36.1	33.1	41.1	
• 5-year survival rate	79.7%	59.7%	69.9%	75.7%	<0.0001[b]
**Advanced stage ORC**					
• Number of patients	8,541	3,374	114	96	
• Median follow-up in months	29.14	20.13	22.59	31.12	
• 5-year survival rate	51.2%	30.7%	38.0%	49.2%	<0.0001[b]
**High grade ORC**					
• Number of patients	10,356	3,374	152	131	
• Median follow-up in months	45.2	34.2	35.1	39.2	
• 5-year survival rate	76.9%	54.3%	71.9%	70.9%	<0.0001[b]
**BrCa**					
• Number of patients	31,871	8,252	401	385	
• Median follow-up in months	48.2	40.2	36.1	41.2	
• 5-year survival rate	86.1%	69.1%	76.5%	79.4%	<0.0001[b]
**Advanced stage[d] BrCa**					
• Number of patients	3,018	1,145	38	47	
• Median follow-up in months	34.1	27.1	32.7	31.2	
• 5-year survival rate	57.9%	36.8%	42.5%	62.2%	<0.0001[b]
**High grade[e] BrCa**					
• Number of patients	7,489	2,266	113	100	
• Median follow-up in months	47.2	39.2	35.1	40.1	
• 5-year survival rate	82.1%	63.5%	74.2%	73.6%	<0.0001[b]
**Right-sided BrCa**					
• Number of patients	15,544	3,990	193	177	
• Median follow-up in months	48.2	40.1	39.1	43.1	
• 5-year survival rate	86.7%	68.9%	84.1%	82.4	<0.0001[b]
**Left-sided BrCa**					
• Number of patients	16,159	4,197	206	208	
• Median follow-up in months	48.1	41.1	33.6	40.6	
• 5-year survival rate	86.1%	70.1%	68.7%	76.7%	<0.0001[b]
**CRC**					
• Number of patients	9,865	3,520	114	109	
• Median follow-up in months	40.1	30.1	34.6	41.1	
• 5-year survival rate	67.4%	49.3%	54.7%	68.8%	<0.0001[b]

**Advanced stage[d] CRC**					
• Number of patients	3,261	1,342	43	29	
• Median follow-up in months	28.1	20.0	17.0	30.1	
• 5-year survival rate	51.3%	31.6%	31.2%	46.5%	<0.0001[b]
**High grade[e] CRC**					
• Number of patients	>1,462	559	23	<11[c]	
• Median follow-up in months	37.1	23.2	46.2	46.2	
• 5-year survival rate	64.3%	39.1%	69.5%	78.7%	<0.0001[b]
**ECa**					
• Number of patients	4,719	1,809	59	67	
• Median follow-up in months	42.2	32.0	26.1	36.1	
• 5-year survival rate	76.1%	53.1%	67.3%	75.9%	<0.0001[b]
**Advanced stage[d] ECa**					
• Number of patients	915	417	12	11	
• Median follow-up in months	29.2	18.1	24.1	36.1	
• 5-year survival rate	48.7%	31.2%	59.2%	23.3%	<0.0001[b]
**High grade[e] ECa**					
• Number of patients	>881	402	<11[c]	15	
• Median follow-up in months	37.2	26.1	23.6	24.1	
• 5-year survival rate	66.6%	36.5%	50.0%	47.2%	<0.0001[b]
**OCa**					
• Number of patients	2,031	658	31	16	
• Median follow-up in months	26.1	13.0	20.1	28.1	
• 5-year survival rate	46.8%	15.2%	49.1%	31.4%	<0.0001[b]

Abbreviations: BrCa, breast cancer; CRC, colorectal cancer; ECa, endometrial cancer; ORC, obesity-related cancers; OCa, ovarian cancer; WLM, weight loss medication.

[a]WLM include Diethylpropion, Liraglutide, Lorcaserin, Orlistat, Phendimetrazine and Phentermine.

[b]Denotes Log-rank test statistical significance at the *p*-value < 0.05 level.

[c]SEER-Medicare data presentation guideline has been followed and all counts less than 11 have been suppressed.

[d]Advanced stage ORC indicated AJCC stage III & IV definition while localized stage ORC indicated stage I & stage II.

[e]High grade ORC indicated G3 (poorly differentiated) and G4 (undifferentiated). Low grade indicated G1(well differentiated) and G2 (moderately differentiated).

Supplemental Table 5. HRs and 95% CIs for the association of pre-diagnostic use of metformin and WLM with all-cause mortality in women ≥65 years old with ORCs.

	**All-cause mortality after IPTW**			
	**Non-Diabetic**		**Diabetic**	
**Category**	**Adjusted HR (95% CI)**	***p*-value**	**Adjusted HR (95% CI)**	***p*-value**
**ORCs cohort**				
• Neither users	Ref		Ref	
• Metformin alone	2.08 (2.01 , 2.17)	<0.0001[a]	1.58 (1.51 , 1.65)	<0.0001[a]
• WLM alone	1.41 (1.35 , 1.47)	<0.0001[a]	2.29 (2.16 , 2.44)	<0.0001[a]
• Dual users	1.79 (1.71 , 1.88)	<0.0001[a]	1.08 (1.01 , 1.15)	0.0160[a]
**Advanced stage ORCs cohort** [b]				
• Neither users	Ref		Ref	
• Metformin alone	1.95 (1.84 , 2.07)	<0.0001[a]	1.38 (1.28 , 1.48)	<0.0001[a]
• WLM alone	1.42 (1.34 , 1.51)	<0.0001[a]	2.49 (2.24 , 2.78)	<0.0001[a]
• Dual users	1.02 (0.92 , 1.12)	0.7547	0.97 (0.88 , 1.08)	0.6042
**High grade ORCs cohort** [c]				
• Neither users	Ref		Ref	
• Metformin alone	2.26 (2.09 , 2.44)	<0.0001[a]	1.58 (1.44 , 1.73)	<0.0001[a]
• WLM alone	1.50 (1.38 , 1.64)	<0.0001[a]	1.69 (1.46 , 1.96)	<0.0001[a]
• Dual users	2.21 (1.98 , 2.47)	<0.0001[a]	1.16 (1.03 , 1.30)	0.0128[a]
**BrCa cohort**				
• Neither users	Ref		Ref	
• Metformin alone	2.20 (2.07 , 2.34)	<0.0001[a]	1.59 (1.48 , 1.71)	<0.0001[a]
• WLM alone	1.85 (1.74 , 1.97)	<0.0001[a]	2.04 (1.86 , 2.24)	<0.0001[a]
• Dual users	1.89 (1.75 , 2.03)	<0.0001[a]	1.21 (1.11 , 1.32)	<0.0001[a]
**Advanced stage BrCa cohort** [b]				
• Neither users	Ref		Ref	
• Metformin alone	2.05 (1.83 , 2.29)	<0.0001[a]	1.25 (1.09 , 1.42)	0.0009[a]
• WLM alone	2.36 (2.08 , 2.67)	<0.0001[a]	0.79 (0.61 , 1.01)	0.0619
• Dual users	1.22 (1.04 , 1.42)	0.0129[a]	0.97 (0.82 , 1.14)	0.6808
**High grade BrCa cohort** [c]				
• Neither users	Ref			
• Metformin alone	2.28 (2.06 , 2.54)	<0.0001[a]	1.40 (1.24 , 1.58)	<0.0001[a]
• WLM alone	1.58 (1.39 , 1.79)	<0.0001[a]	1.89 (1.58 , 2.27)	<0.0001[a]
• Dual users	1.73 (1.47 , 2.03)	<0.0001[a]	1.26 (1.08 , 1.46)	0.0023[a]
**Right-sided BrCa cohort**				
• Neither users	Ref		Ref	
• Metformin alone	2.48 (2.27 , 2.69)	<0.0001[a]	1.59 (1.44 , 1.76)	<0.0001[a]
• WLM alone	1.58 (1.43 , 1.74)	<0.0001[a]	2.11 (1.79 , 2.48)	<0.0001[a]
• Dual users	2.10 (1.86 , 2.37)	<0.0001[a]	1.17 (1.04 , 1.33)	0.0121[a]
**Left-sided BrCa cohort**				
• Neither users	Ref		Ref	
• Metformin alone	1.97 (1.80 , 2.15)	<0.0001[a]	1.59 (1.44 , 1.75)	<0.0001[a]
• WLM alone	2.34 (2.14 , 2.55)	<0.0001[a]	2.24 (1.99 , 2.51)	<0.0001[a]
• Dual users	2.06 (1.87 , 2.28)	<0.0001[a]	1.38 (1.21 , 1.56)	<0.0001[a]
**CRC cohort**				
• Neither users	Ref		Ref	
• Metformin alone	1.81 (1.69 , 1.94)	<0.0001[a]	1.41 (1.29 , 1.52)	<0.0001[a]

• WLM alone	1.18 (1.09 , 1.27)	<0.0001[a]	2.69 (2.42 , 2.99)	<0.0001[a]
• Dual users	1.96 (1.79 , 2.14)	<0.0001[a]	0.93 (0.84 , 1.05)	0.2739
**Advanced stage CRC cohort [b]**				
• Neither users	Ref		Ref	
• Metformin alone	1.91 (1.74 , 2.11)	<0.0001[a]	1.44 (1.27 , 1.62)	<0.0001[a]
• WLM alone	1.62 (1.47 , 1.78)	<0.0001[a]	4.04 (3.45 , 4.72)	<0.0001[a]
• Dual users	2.81 (2.34 , 3.37)	<0.0001[a]	1.21 (1.03 , 1.43)	0.0208[a]
**High grade CRC cohort [c]**				
• Neither users	Ref		Ref	
• Metformin alone	2.88 (2.43 , 3.43)	<0.0001[a]	1.69 (1.38 , 2.08)	<0.0001[a]
• WLM alone	2.44 (2.07 , 2.88)	<0.0001[a]	NC	NC
• Dual users	6.06 (4.71 , 7.81)	<0.0001[a]	0.43 (0.28 , 0.65)	<0.0001[a]
**ECa cohort**				
• Neither users	Ref		Ref	
• Metformin alone	2.45 (2.19 , 2.73)	<0.0001[a]	1.81 (1.59 , 2.06)	<0.0001[a]
• WLM alone	0.87 (0.75 , 1.02)	0.0839	2.35 (1.94 , 2.86)	<0.0001[a]
• Dual users	1.18 (0.97 , 1.44)	0.0943	1.19 (1.01 , 1.41)	0.0347[a]
**Advanced stage ECa cohort [b]**				
• Neither users	Ref		Ref	
• Metformin alone	1.91 (1.60 , 2.28)	<0.0001[a]	1.87 (1.48 , 2.38)	<0.0001[a]
• WLM alone	0.05. (0.03 , 0.09)	<0.0001[a]	2.92 (1.66 , 5.16)	0.0002[a]
• Dual users	NC	NC	1.09 (0.75 , 1.60)	0.6309
**High grade ECa cohort [c]**				
• Neither users	Ref		Ref	
• Metformin alone	2.13 (1.70 , 2.67)	<0.0001[a]	2.07 (1.61 , 2.67)	<0.0001[a]
• WLM alone	0.96 (0.58 , 1.57)	0.8623	2.11 (1.33 , 3.33)	0.0014[a]
• Dual users	4.39 (3.22 , 6.01)	<0.0001[a]	1.51 (1.09 , 2.07)	0.0111[a]
**OCa cohort**				
• Neither users	Ref		Ref	
• Metformin alone	2.21 (1.98 , 2.48)	<0.0001[a]	1.79 (1.53 , 2.09)	<0.0001[a]
• WLM alone	1.19 (1.05 , 1.35)	0.0057[a]	3.47 (2.45 , 4.92)	<0.0001[a]
• Dual users	2.19 (1.85 , 2.59)	<0.0001[a]	1.47 (1.13 , 1.93)	0.0046[a]

Abbreviations: aHR, adjusted hazard ratio; BrCa, breast cancer; CI, confidence interval; CRC, colorectal cancer; EC, endometrial cancer; NC, not calculated; OCa, ovarian cancer; ORC, obesity-related cancers; WLM, weight loss medication.

Multivariable Cox regressions models are adjusted for age at prescription, race/ethnicity, cancer stage, cancer grade, Charlson comorbidity index, diabetes, use of insulin, hypertension, hyperlipidemia, cardiovascular disease, malaise and fatigue, muscular wasting and atrophy, hypogonadism, anterior pituitary dysfunction, depression, osteoporosis, Cushing syndrome, hypothyroidism, hyperthyroidism, polycystic ovary syndrome, poverty rate.

[a]Denotes statistical significance at the *p*-value < 0.05 level.

[b]Advanced stage ORC indicated AJCC stage III & IV definition while localized stage ORC indicated stage I & stage II.

[c]High grade ORC indicated G3 (poorly differentiated) and G4 (undifferentiated). Low grade indicated G1(well differentiated) and G2 (moderately differentiated).

Supplemental Table 6. HRs and 95% CIs for the association of pre-diagnostic use of metformin and WLM with cancer-specific mortality in women ≥65 years old with ORCs.

	Cancer-specific mortality after IPTW			
	**Non-Diabetic**		**Diabetic**	
**Category**	**Adjusted HR (95% CI)**	***p*-value**	**Adjusted HR (95% CI)**	***p*-value**
**ORCs cohort**				
• Neither users	Ref		Ref	
• Metformin alone	1.89 (1.79 , 1.99)	<0.0001[a]	1.51 (1.41 , 1.61)	<0.0001[a]
• WLM alone	1.29 (1.22 , 1.38)	<0.0001[a]	2.45 (2.24 , 2.68)	<0.0001[a]
• Dual users	1.50 (1.40 , 1.60)	<0.0001[a]	1.14 (1.04 , 1.25)	0.0045[a]
**Advanced stage ORCs cohort [b]**				
• Neither users	Ref		Ref	
• Metformin alone	1.87 (1.75 , 1.99)	<0.0001[a]	1.38 (1.26 , 1.51)	<0.0001[a]
• WLM alone	1.55 (1.45 , 1.66)	<0.0001[a]	3.28 (2.91 , 3.69)	<0.0001[a]
• Dual users	0.75 (0.66 , 0.84)	<0.0001[a]	1.10 (0.98 , 1.24)	0.1045
**High grade ORCs cohort [c]**				
• Neither users	Ref		Ref	
• Metformin alone	2.12 (1.92 , 2.35)	<0.0001[a]	1.59 (1.39 , 1.82)	<0.0001[a]
• WLM alone	1.51 (1.36 , 1.67)	<0.0001[a]	2.27 (1.86 , 2.78)	<0.0001[a]
• Dual users	3.94 (3.50 , 4.44)	<0.0001[a]	1.22 (1.03 , 1.44)	0.0221[a]
**BrCa cohort**				
• Neither users	Ref		Ref	
• Metformin alone	2.12 (1.93 , 2.33)	<0.0001[a]	1.65 (1.46 , 1.85)	<0.0001[a]
• WLM alone	1.24 (1.11 , 1.38)	0.0001[a]	1.82 (1.50 , 2.19)	<0.0001[a]
• Dual users	1.29 (1.15 , 1.46)	<0.0001[a]	1.22 (1.06 , 1.42)	0.0068[a]
**Advanced stage BrCa cohort [b]**				
• Neither users	Ref		Ref	
• Metformin alone	1.94 (1.68 , 2.24)	<0.0001[a]	1.49 (1.25 , 1.77)	<0.0001[a]
• WLM alone	1.61 (1.38 , 1.87)	<0.0001[a]	1.01 (0.76 , 1.33)	0.9735
• Dual users	1.21 (1.01 , 1.46)	0.0427[a]	1.47 (1.20 , 1.79)	0.0002[a]
**High grade BrCa cohort [c]**				
• Neither users	Ref		Ref	
• Metformin alone	1.98 (1.69 , 2.32)	<0.0001[a]	1.48 (1.22 , 1.79)	<0.0001[a]
• WLM alone	1.08 (0.89 , 1.29)	0.6063	2.93 (2.21 , 3.90)	<0.0001[a]
• Dual users	3.86 (3.25 , 4.58)	<0.0001[a]	1.77 (1.43 , 2.19)	<0.0001[a]
**Right-sided BrCa cohort**				
• Neither users	Ref		Ref	
• Metformin alone	2.38 (2.08 , 2.73)	<0.0001[a]	1.61 (1.32 , 1.95)	<0.0001[a]
• WLM alone	1.04 (0.88 , 1.23)	0.6582	4.95 (3.89 , 6.30)	<0.0001[a]
• Dual users	0.67 (0.57 , 0.79)	<0.0001[a]	1.53 (1.25 , 1.88)	<0.0001[a]
**Left-sided BrCa cohort**				
• Neither users	Ref		Ref	
• Metformin alone	1.90 (1.65 , 2.19)	<0.0001[a]	1.76 (1.47 , 2.11)	<0.0001[a]
• WLM alone	1.42 (1.22 , 1.65)	<0.0001[a]	0.98 (0.74 , 1.29)	0.8866
• Dual users	2.09 (1.77 , 2.45)	<0.0001[a]	1.03 (0.79 , 1.34)	0.8380
**CRC cohort**				
• Neither users	Ref		Ref	
• Metformin alone	1.68 (1.54 , 1.83)	<0.0001[a]	1.29 (1.15 , 1.45)	<0.0001[a]

• WLM alone	1.47 (1.35 , 1.59)	<0.0001[a]	3.14 (2.78 , 3.56)	<0.0001[a]
• Dual users	1.44 (1.27 , 1.63)	<0.0001[a]	1.37 (1.17 , 1.59)	<0.0001[a]
**Advanced stage CRC cohort** [b]				
• Neither users	Ref		Ref	
• Metformin alone	1.76 (1.58 , 1.96)	<0.0001[a]	1.37 (1.17 , 1.59)	<0.0001[a]
• WLM alone	1.96 (1.78 , 2.16)	<0.0001[a]	4.51 (3.83 , 5.31)	<0.0001[a]
• Dual users	1.33 (1.06 , 1.66)	0.0123[a]	1.37 (1.12 , 1.69)	0.0024[a]
**High grade CRC cohort** [c]				
• Neither users	Ref		Ref	
• Metformin alone	3.11 (2.52 , 3.85)	<0.0001[a]	1.34 (1.03 , 1.75)	0.0323[a]
• WLM alone	3.33 (2.76 , 4.02)	<0.0001[a]	NC	NC
• Dual users	7.39 (5.48 , 9.97)	<0.0001[a]	NC	NC
**ECa cohort**				
• Neither users	Ref		Ref	
• Metformin alone	2.04 (1.77 , 2.35)	<0.0001[a]	1.59 (1.32 , 1.92)	<0.0001[a]
• WLM alone	0.25 (0.18 , 0.34)	<0.0001[a]	1.43 (1.13 , 1.81)	0.0027[a]
• Dual users	1.50 (1.27 , 1.77)	<0.0001[a]	0.76 (0.59 , 0.97)	0.0261[a]
**Advanced stage ECa cohort** [b]				
• Neither users	Ref		Ref	
• Metformin alone	1.99 (1.65 , 2.41)	<0.0001[a]	1.39 (1.05 , 1.86)	0.0211[a]
• WLM alone	0.05 (0.03 , 0.10)	<0.0001[a]	2.11 (1.09 , 4.07)	0.0258[a]
• Dual users	NC	NC	0.40 (0.26 , 0.63)	<0.0001[a]
**High grade ECa cohort** [c]				
• Neither users	Ref		Ref	
• Metformin alone	2.26 (1.69 , 3.02)	<0.0001[a]	1.78 (1.26 , 2.51)	0.0011[a]
• WLM alone	1.07 (0.70 , 1.63)	0.7455	2.13 (1.29 , 3.51)	0.0031[a]
• Dual users	2.66 (1.97 , 3.59)	<0.0001[a]	0.16 (0.09 , 0.27)	<0.0001[a]
**OCa cohort**				
• Neither users	Ref		Ref	
• Metformin alone	1.85 (1.63 , 2.11)	<0.0001[a]	1.63 (1.36 , 1.95)	<0.0001[a]
• WLM alone	1.46 (1.28 , 1.66)	<0.0001[a]	4.02 (3.01 , 5.37)	<0.0001[a]
• Dual users	2.46 (2.12 , 2.86)	<0.0001[a]	1.46 (1.11 , 1.93)	0.0076[a]

Abbreviations: aHR, adjusted hazard ratio; BrCa, breast cancer; CI, confidence interval; CRC, colorectal cancer; EC, endometrial cancer; NC, not calculated; OCa, ovarian cancer; ORC, obesity-related cancers; WLM, weight loss medication.

Fine-Gray competing risks (cancer-specific mortality) models are adjusted for age at prescription, race/ethnicity, cancer stage, cancer grade, Charlson comorbidity index, diabetes, use of insulin, hypertension, hyperlipidemia, cardiovascular disease, malaise and fatigue, muscular wasting and atrophy, hypogonadism, anterior pituitary dysfunction, depression, osteoporosis, Cushing syndrome, hypothyroidism, hyperthyroidism, polycystic ovary syndrome, poverty rate.

[a]Denotes statistical significance at the *p*-value < 0.05 level.

[b]Advanced stage ORC indicated AJCC stage III & IV definition while localized stage ORC indicated stage I & stage II.

[c]High grade ORC indicated G3 (poorly differentiated) and G4 (undifferentiated). Low grade indicated G1(well differentiated) and G2 (moderately differentiated).

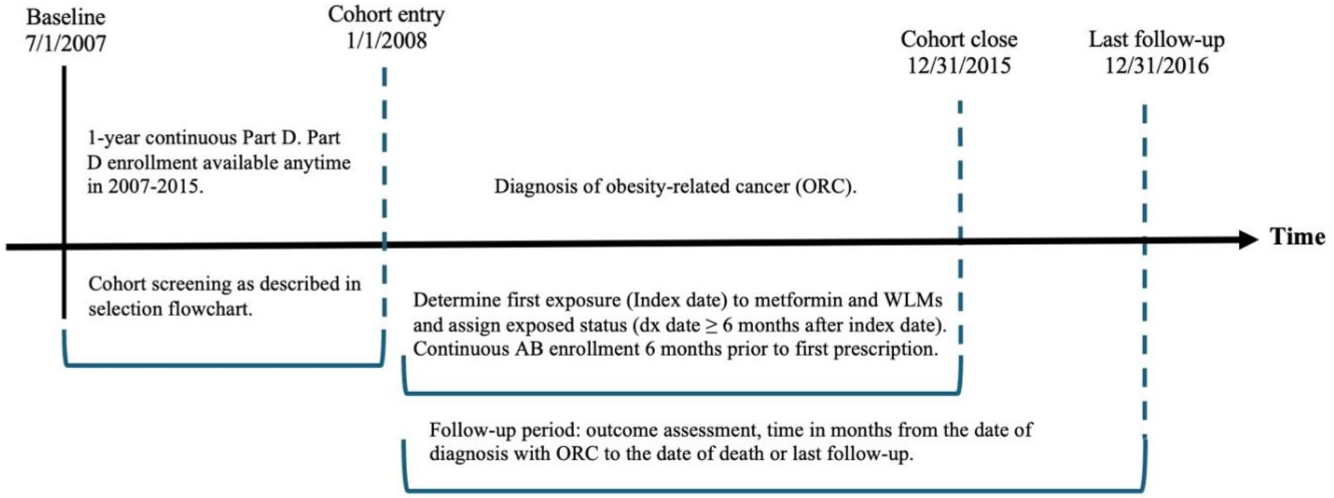

Supplemental Fig. 1. Study's timeline.

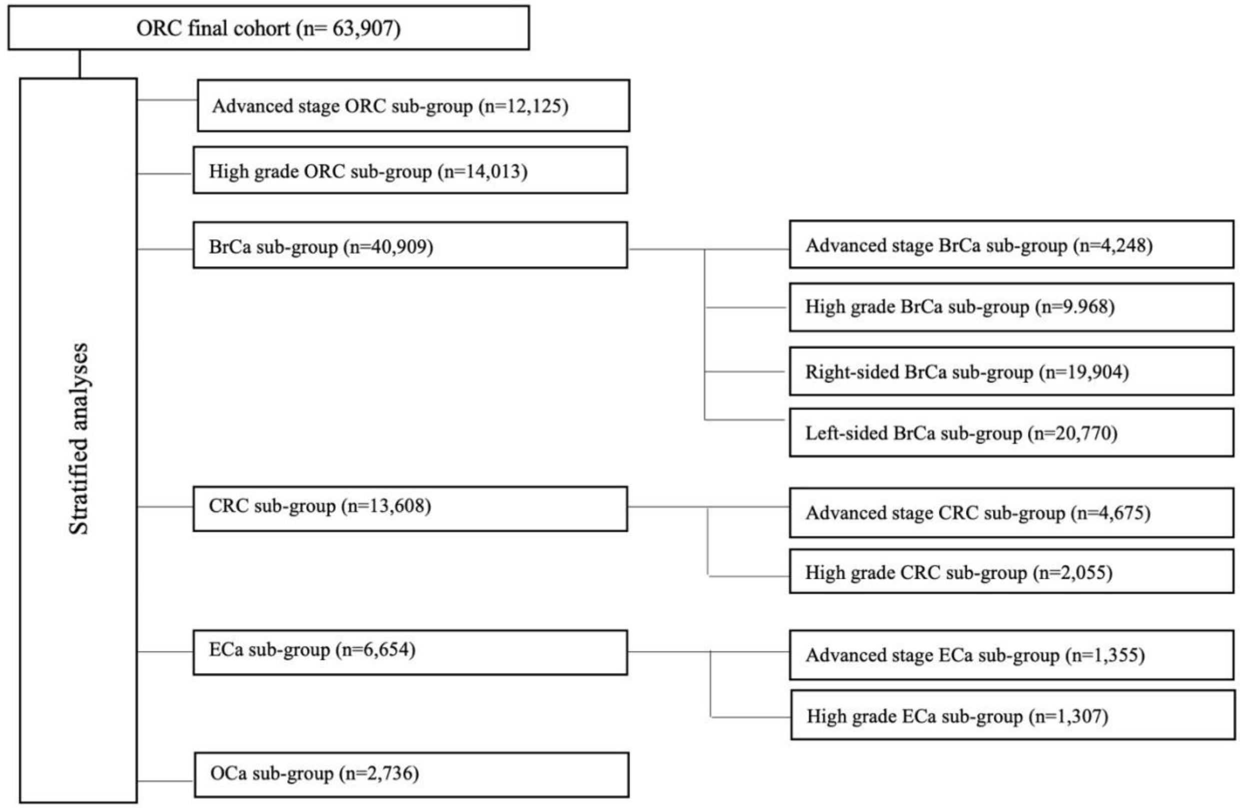

Supplemental Fig. 2. Obesity-related cancer (ORC) subgroups.

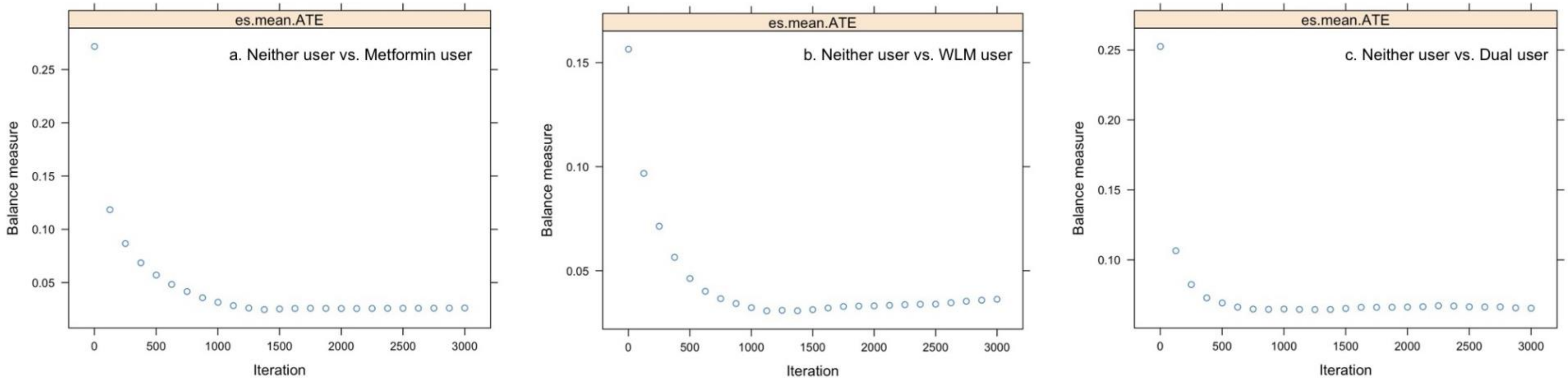

Supplemental Fig. 3. Inverse probability of treatment weighting (IPTW) using Propensity Score Diagnostics: Convergence of the gradient boosting algorithm assessment. The algorithm in figures S3 a, b and c reached stable solutions within a sufficient and reasonable number of iterations. They were optimized at 1393 iterations for metformin, 1146 iterations for WLM, and 1230 iterations for dual user, suggesting that the models converged appropriately.

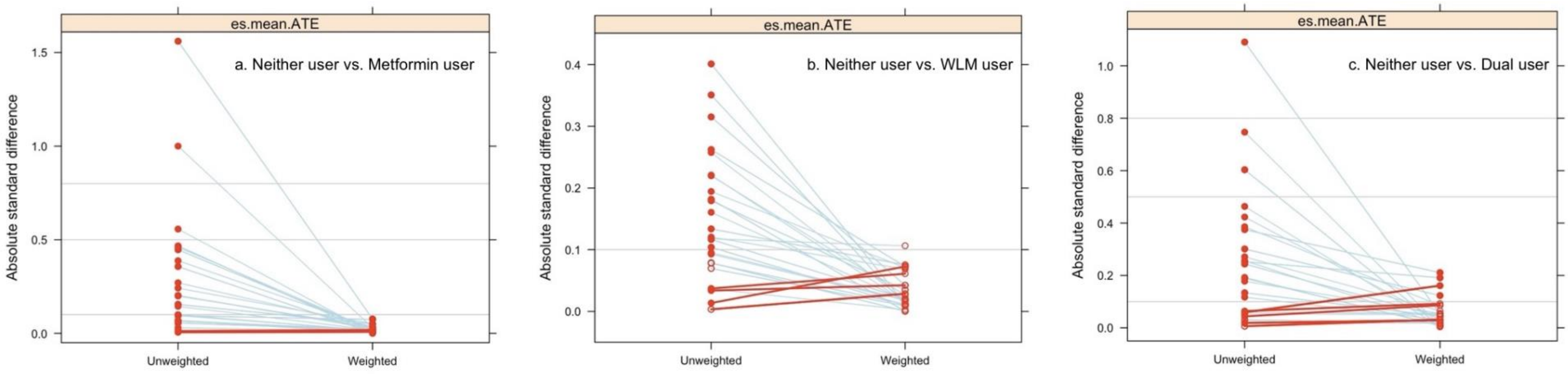

Supplemental Fig. 4. Inverse probability of treatment weighting (IPTW) using Propensity Score Diagnostics: t-test p-values for weighted pretreatment variables. As shown in figures S5 a, b and c, before weighting (closed circles), the groups have statistically significant differences on many variables (*p*-values are near zero). After IPW (open circles) the p-values are generally running near or above the diagonal line, suggesting that the balance was likely achieved.

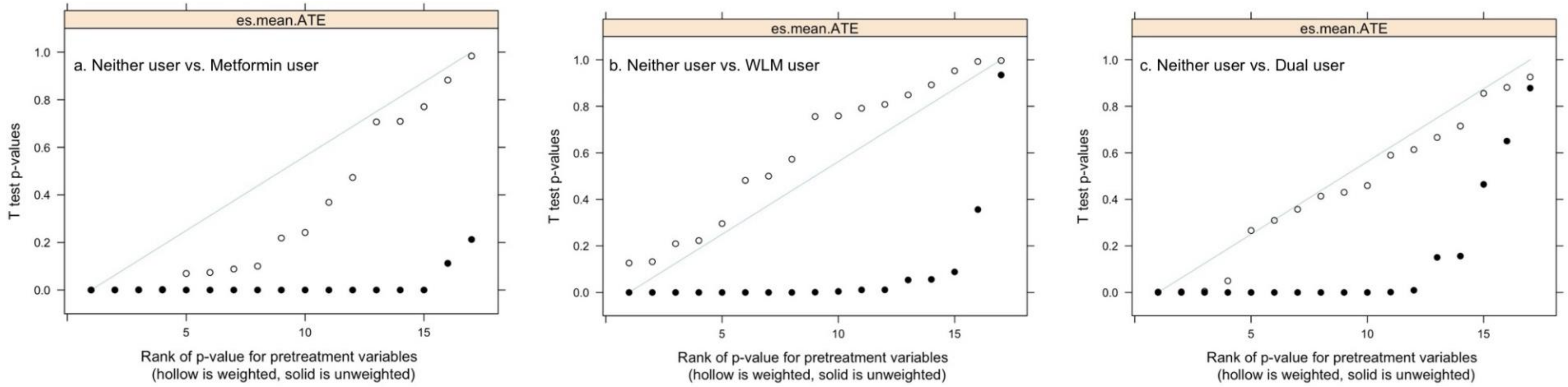

Supplemental Fig. 5. Inverse probability of treatment weighting (IPTW) using Propensity Score Diagnostics: t-test p-values for weighted pretreatment variables. As shown in figures S5 a, b and c, before weighting (closed circles), the groups have statistically significant differences on many variables (*p*-values are near zero). After IPW (open circles) the p-values are generally running near or above the diagonal line, suggesting that the balance was likely achieved.
